# NINJ1 regulates ferroptosis via xCT antiporter interaction and CoA modulation

**DOI:** 10.1038/s41419-024-07135-1

**Published:** 2024-10-18

**Authors:** Ssu-Yu Chen, Jianli Wu, Yubin Chen, Ya-En Wang, Yasaman Setayeshpour, Chiara Federico, Alexander A. Mestre, Chao-Chieh Lin, Jen-Tsan Chi

**Affiliations:** 1 Department of Pharmacology and Cancer Biology, Duke University School of Medicine, Durham, NC 27710 USA; 2 Department of Molecular Genetics and Microbiology, Duke University School of Medicine, Durham, NC 27710 USA; 3 Department of Biochemistry, Duke University School of Medicine, Durham, NC 27710 USA; 4 Center for Advanced Genomic Technologies, Duke University School of Medicine, Durham, NC 27710 USA

**Keywords:** Cancer, Molecular biology, Cell death

## Abstract

Ninjurin-1 (NINJ1), initially identified as a stress-induced protein in neurons, recently emerged as a key mediator of plasma membrane rupture (PMR) during apoptosis, necrosis, and pyroptosis. However, its involvement in ferroptosis is less well elucidated. Here, we demonstrate that NINJ1 also plays a crucial role in ferroptosis, but through a distinct mechanism. NINJ1 knockdown significantly protected cancer cells against ferroptosis induced only by xCT inhibitors but no other classes of ferroptosis-inducing compounds (FINs). Glycine, known to inhibit canonical NINJ1-mediated membrane rupture in other cell deaths, had no impact on ferroptosis. A compound screen revealed that the ferroptosis protective effect caused by NINJ1 knockdown can be abolished by pantothenate kinase inhibitor (PANKi), buthionine sulfoximine (BSO), and diethylmaleate (DEM). These results suggest that this ferroptosis protection is mediated via Coenzyme A (CoA) and glutathione (GSH), both of which were found to be elevated upon NINJ1 knockdown. Furthermore, we discovered that NINJ1 interacts with the xCT antiporter, which is responsible for cystine uptake for the biosynthesis of CoA and GSH. The removal of NINJ1 increased xCT levels and stability, enhancing cystine uptake and thereby providing protection against ferroptosis. Conversely, NINJ1 overexpression reduced xCT levels and sensitized ferroptosis. These findings reveal that NINJ1 regulates ferroptosis via a non-canonical mechanism, distinct from other regulated cell deaths.

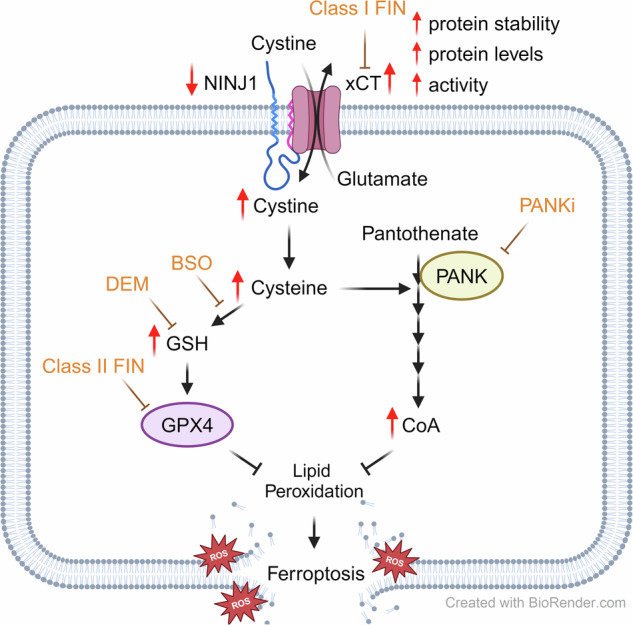

## Introduction

NINJ1 was initially discovered due to its induction upon nerve damage and found to promote neurite regeneration [1]. Further studies revealed its involvement in cell adhesion [2], migration, and inflammation processes [3]. Structurally, NINJ1 is a homophilic type II transmembrane protein conserved across species [4, 5]. With extracellular and cytoplasmic domains, NINJ1 acts as a molecular bridge to transmit signals across the cell membrane to influence cell behavior in response to external stimuli. The role of NINJ1 in cancer is multifaceted. Some studies associate elevated NINJ1 levels with enhanced tumor progression and mobility [6, 7]. Other research suggests that NINJ1 overexpression suppresses tumor growth [8–10] and its expression is downregulated during tumor recurrence and treatment resistance [11]. This observation aligns with the involvement of NINJ1 in various cell death processes [4]. Consequently, NINJ1 has been suggested as a biomarker and a therapeutic target in multiple human cancers.

Cell death plays a crucial role in both biological growth and disease pathophysiology [12]. Most regulated types of cell death involve protein pores disrupting osmotic balance, leading to water influx, cell swelling, and eventual cell membrane rupture. Different types of cell death involve different proteins, including the BCL family in apoptosis [13], gasdermin D (GSDMD) in pyroptosis [14], and Mixed Lineage Kinase Domain-Like Protein (MLKL) in necroptosis [15], respectively. Interestingly, the NINJ1 protein was recently found to be involved in all three types of lytic cell death by aggregation to form a protein pore mediating the PMR [4, 16]. The application of neutralizing antibodies targeting NINJ1 reduced cell death and prevented various tissue injuries [17]. Additionally, glycine inhibits NINJ1 aggregation and blocks these types of cell death [18]. These studies have firmly established NINJ1 as a critical and common executor of multiple cell death mechanisms by aggregating to form membrane-rupturing protein pores. However, the role of NINJ1 in ferroptosis was not discussed in these studies.

Ferroptosis is a regulated form of cell death characterized by iron dependency, lipid peroxidation, and oxidative stress [19, 20]. Ferroptosis is suppressed by multiple endogenous antioxidant pathways, including System Xc-GSH-GPX4, DHODH-CoQH2 pathway, and mevalonate pathway [21, 22]. Inhibition of these protective mechanisms leads to iron-catalyzed oxidative damage of lipid membranes and subsequent cell rupture, resulting in ferroptosis. Therefore, ferroptosis can be induced by diverse FINs, classified into distinct classes according to their targets and mechanisms. Class I (e.g., erastin, sulfasalazine (SAS)) targets xCT and cystine uptake [19, 23, 24], Class II (e.g., RSL3, ML162) targets GPX4 enzymatic activity [25], Class III (e.g., FIN56) depletes GPX4 and ubiquinone [26], and Class IV (e.g., FINO2) indirectly inhibits GPX4 while directly oxidizing iron [27–29]. Ferroptosis holds significant implications for human diseases [21, 22], particularly in cancer, where it serves as a critical tumor suppression mechanism. Since many treatment-resistant tumor cells have been found to be highly sensitive to ferroptosis [30, 31], the induction of ferroptosis has emerged as a promising therapeutic strategy [32].

While the involvement of NINJ1 in various types of cell death is well-established [4], its role in ferroptosis is less well elucidated. Recent studies demonstrated that NINJ1 deficiency failed to confer protection against ferroptotic cell death induced by RSL3 and ML162, GPX4 inhibitors, in RAW 264.7 cells and murine embryonic fibroblasts (MEFs) [33, 34]. Additionally, a previous paper indicated that NINJ1 deletion in mouse bone marrow-derived macrophages (BMDMs) or MEFs conferred prolonged protection against cell lysis, as measured by LDH release, but it did not prevent cell death, as determined by ATP production. They also stated, “*absence of NINJ1 had no impact on the early events that are observable in ferroptotic cells, such as lipid peroxidation, calcium influx, or cell swelling*” [35]. However, this previous study did not offer any explanation for the discrepancy.

In this study, we systemically examined the role of NINJ1 in the ferroptosis of cancer cells induced by various FINs. While we were able to reproduce the finding that NINJ1 knockdown did not affect RSL3-induced ferroptosis, we found that NINJ1 knockdown robustly protected cancer cells against ferroptosis induced by xCT inhibitors, such as erastin and SAS. We also observed that this protection against ferroptosis can be abolished by the chemical inhibition of the biosynthesis of either CoA (PANKi) or GSH (BSO and DEM), indicating the important role of both ferroptosis-protecting branches. Furthermore, we found that NINJ1 knockdown significantly elevated intracellular CoA and GSH levels. CoA, similar to NINJ1 knockdown, protected ferroptosis triggered by xCT inhibitors while showing no protection against GPX4 inhibitors [24, 36, 37]. GSH, a well-known protector against ferroptosis, serves as a cofactor for GPX4. Additionally, we detected a physical association between NINJ1 and xCT. NINJ1 knockdown increased xCT stability, expression, and functionality, enhancing cystine uptake and increasing the CoA and GSH levels. Conversely, NINJ1 overexpression specifically enhanced ferroptosis by downregulating the levels and activities of xCT. These findings suggest that NINJ1 may have therapeutic potential in cancer treatment by regulating ferroptosis.

## Results

### NINJ1 knockdown protected against ferroptosis specifically triggered by class I FINs

**Fig. 1:**
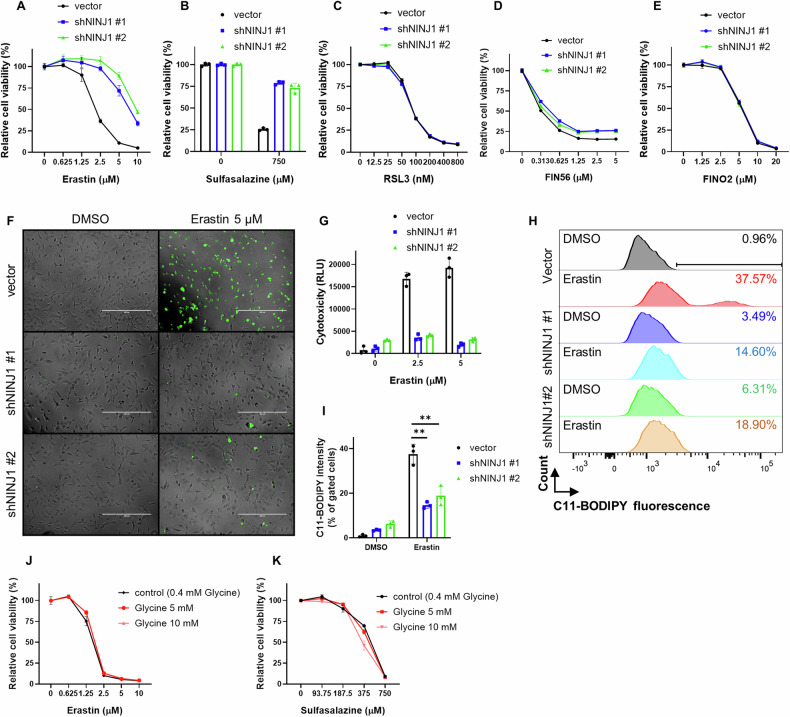
NINJ1 knockdown protected against ferroptosis specifically triggered by class I FINs. **A**–**E** Cell viability of HT-1080 cells, transduced with a control vector or two NINJ1-targeting shRNA, was determined by CellTiter-Glo assay following treatment with indicated concentrations of (**A**) erastin (23 h), (**B**) sulfasalazine (28 h), (**C**) RSL3 (19 h), (**D**) FIN56 (19 h), and (**E**) FINO2 (19 h). **F**-**G** NINJ1 knockdown abolished erastin-induced membrane rupture in HT-1080 cells. Membrane rupture of control and NINJ1-knockdown HT-1080 cells were observed by CellTox Green under fluorescence microscopy (**F**) and quantified (**G**) following erastin treatment (2.5 and 5 μM, 22 h). Scale bar: 400 μm. **H**-**I** NINJ1 knockdown inhibited erastin-induced lipid peroxidation in HT-1080 cells. Lipid peroxidation of control and NINJ1-knockdown HT-1080 cells were determined by C11-BODIPY staining (**H**) and quantified by % of lipid peroxidation positive cells (**I**) following erastin treatment (2.5 μM, 20 h). **J**-**K** Cell viability of HT-1080 cells was determined by CellTiter-Glo assay following treatment with indicated concentrations of (**J**) erastin (24 h) or (**K**) sulfasalazine (48 h) combined with glycine (5 or 10 mM). Error bars in (**A**–**E**), (**G**), (**I**), and (**J**-**K**) represent SEM (*n* = 3+).

To systemically investigate the role of NINJ1 in ferroptosis, NINJ1 expression was successfully knocked down by two independent shRNA in HT-1080 human fibrosarcoma cells (Supplemental Fig. 1A). NINJ1-knockdown and control (empty vector) cells were then exposed to a dose-titration of various classes of FINs. The sensitivity of these cells to ferroptosis was then compared by measuring ATP levels using the CellTiter-Glo assay. We found that NINJ1 knockdown robustly protected HT-1080 cells against ferroptosis induced by class I FINs, such as erastin and SAS (Fig. 1A, B). Conversely, NINJ1 knockdown did not affect ferroptosis triggered by class II FIN (RSL3) (Fig. 1C), which aligns with recent studies demonstrating the dispensability of NINJ1 in RSL3-induced ferroptotic cell death [33–35]. Similarly, NINJ1 knockdown did not affect ferroptosis induced by class III (FIN56) or class IV FINs (FINO2) (Fig. 1D, E). These results indicate that NINJ1 regulates ferroptosis specifically induced by xCT inhibition. Consistent with these phenotypes, NINJ1 knockdown also inhibited other erastin-induced molecular features of ferroptosis, including membrane rupture (Fig. 1F, G) and lipid peroxidation (Fig. 1H, I). A previous study showed that glycine protected cell death, such as pyroptosis, necrosis, and apoptosis, by inhibiting NINJ1 aggregation [18]. However, our findings indicate glycine did not protect against ferroptosis (Fig. 1J, K), suggesting NINJ1 aggregation might not contribute to the ferroptosis-protective effects induced by NINJ1 knockdown. We also validated the ferroptosis-protective effects of NINJ1 knockdown in additional cancer cell types, including—MDA-MB-231 (breast cancer) and PC3 cells (prostate cancer) (Supplemental Fig. 1B–I). Collectively, these results suggest that NINJ1 knockdown specifically protects cancer cells against class I FINs, but not other classes of FINs.

### NINJ1 knockdown-mediated ferroptosis protection is abolished by the inhibition of CoA and GSH synthesis

**Fig. 2:**
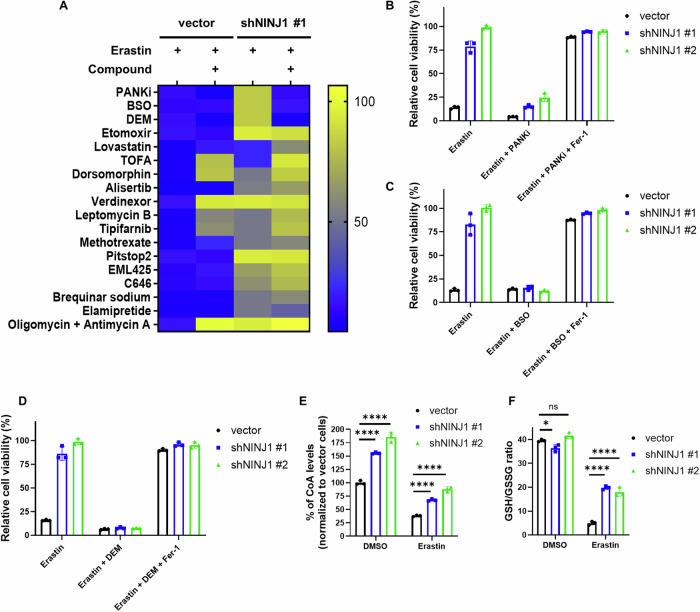
NINJ1 knockdown-mediated ferroptosis protection is abolished by the inhibition of CoA and GSH synthesis. **A** The heatmap showed the relative cell viability (% of DMSO- or compound-treated cells under erastin treatment) of control and NINJ1-knockdown HT-1080 cells, as summarized from the cell viability graphs in Fig. 2B–D and supplemental Fig. 2. Each cell viability graph was generated individually using the CellTiter-Glo assay following treatment with the indicated concentrations of erastin, with or without the specified compounds. The relative viability percentages for cells treated with 5 μM erastin, combined with or without the indicated compounds, were then compiled into the heatmap. **B**–**D** Cell viability of control and NINJ1-knockdown HT-1080 cells were determined by CellTiter-Glo assay following treatment with erastin (5 μM) and ferrostatin-1 (Fer-1, 10 μM), with or without either (**B**) pantothenate kinase inhibitor (PANKi, 5 μM), (**C)** buthionine sulfoximine (BSO, 500 μM), or (**D**) diethylmaleate (DEM, 200 μM) for 24 h. **E** Intracellular CoA levels of control and NINJ1-knockdown HT-1080 cells following erastin treatment (1.25 μM, 24 h) were determined by Coenzyme A Assay Kit. **F** The GSH/GSSG ratio of control and NINJ1-knockdown HT-1080 cells following erastin treatment (1.25 μM, 24 h) were measured by the GSH/GSSG-Glo Assay. Error bars in (**B**–**F**) represent SEM (*n* = 3+).

The specificity of the NINJ1 knockdown protection phenotype against class I FINs suggested a mechanism upstream of the lipid repair enzyme GPX4. To elucidate the potential mechanisms underlying this protection phenotype, we adopted a chemical genetic approach by conducting a focused compound screen that included compounds targeting ferroptosis-related pathways. Our goal was identifying compounds capable of abolishing this ferroptosis protection by reversing the relevant mechanisms. This involved treating both control and NINJ1 knockdown cells with DMSO or erastin, either alone or in combination with various compounds. The relative cell viability, expressed as a percentage of DMSO- or compound-treated cells under erastin treatment, is summarized as a heatmap (Fig. 2A), with detailed results for individual compounds presented in Fig. 2B–D and Supplemental Fig. 2. The examined compounds included etomoxir [38] (β-oxidation inhibitor, Supplemental Fig. 2A), lovastatin [26] (3-hydroxy-3-methylglutaryl-CoA reductase (HMGCR) inhibitor, Supplemental Fig. 2B), TOFA [39] (acetyl-CoA carboxylase (ACC) inhibitor, Supplemental Fig. 2C), dorsomorphin [39] (AMPK inhibitor, Supplemental Fig. 2D), alisertib [40, 41] (Aurora kinase A inhibitor, Supplemental Fig. 2E), verdinexor and leptomycin B [42] (XPO1/CRM1 inhibitors, Supplemental Fig. 2F–G), tipifarnib [43] (farnesyltransferase inhibitor, Supplemental Fig. 2H), methotrexate [44] (dihydrofolate reductase inhibitor, Supplemental Fig. 2I), pitstop2 [45] (clathrin-mediated endocytosis inhibitor, Supplemental Fig. 2J), EML425 and C646 [46] (p300/CBP Inhibitor, Supplemental Fig. 2K–L), brequinar [47] (dihydroorotate dehydrogenase (DHODH) inhibitors, Supplemental Fig. 2M), elamipretide [48] (mitochondrial-targeted peptide, Supplemental Fig. 2N), and the combination treatment of oligomycin and antimycin A [49] (mitophagy induction, Supplemental Fig. 2O). Remarkably, among all the tested compounds, only three compounds, including PANKi, BSO, and DEM, effectively abolished the observed ferroptosis protection mediated by NINJ1 knockdown (Fig. 2A–D). We further validated this reduction in ferroptosis protection across all tested NINJ1-targeting shRNAs. Additionally, this sensitizing effect could be reversed by ferrostatin-1, further confirming the role of ferroptosis in the resensitization to erastin (Fig. 2B–D).

Pantothenate kinase (PANK) mediates the first and rate-limiting step of the CoA de novo biosynthesis [50]. Therefore, we evaluated intracellular CoA levels in HT-1080 cells following NINJ1 knockdown. Surprisingly, NINJ1 knockdown significantly elevated intracellular CoA levels both before and after erastin treatment (Fig. 2E). Additionally, PANKi significantly abolished the enhanced CoA levels induced by NINJ1 knockdown in erastin-treated cells, while having relatively modest effects on the GSH/GSSG ratio. (Supplemental Fig. 3A, B). NINJ1 knockdown also led to modest upregulation of PANK1 expression (Supplemental Fig. 3G–I), potentially contributing to increased CoA synthesis. Previous research has demonstrated the ability of CoA to rescue ferroptosis induced by xCT inhibition [24, 36]. Results from our own separate studies, currently under review [37], also show that CoA addition rescues ferroptosis triggered by class I FIN but no other classes of FINs, consistent with the pattern of ferroptosis associated with NINJ1 knockdown. These findings suggest that increased CoA levels may play a role in the ferroptosis protection observed with NINJ1 knockdown.

The other two compounds found to mitigate ferroptosis protection were BSO and DEM. BSO inhibits γ-glutamylcysteine synthetase, a crucial enzyme mediating GSH synthesis, impairing GSH production [51]. DEM is an electrophilic reagent that forms a conjugate with GSH, leading to rapid GSH depletion and increased cystine import [52–55]. Given these two compounds can abolish the protection phenotypes, we assessed GSH levels following NINJ1 knockdown. Although NINJ1 knockdown did not induce a significant alteration in the GSH/GSSG ratio without erastin, a noticeable increase in the GSH/GSSG ratio was observed in the NINJ1 knockdown group after erastin treatment (Fig. 2F). This increase was significantly abolished when BSO and DEM were combined with erastin (Supplemental Fig. 3D and F). Surprisingly, combining BSO and DEM with erastin also affected the elevated CoA levels induced by NINJ1 knockdown (Supplemental Fig. 3C and E). This effect may be due to the already limited cysteine availability and increased oxidative stress following erastin treatment [55, 56], or it could involve additional regulatory mechanisms that remain to be determined. Consistently, our own separate study also demonstrated that combining BSO treatment with PANKi or COASY knockdown, both of which inhibit CoA synthesis, led to significant cell death in HT-1080 cells [37]. Notably, this cell death could be rescued by the addition of CoA [37], underscoring the critical role of CoA levels, alongside GSH levels, in the regulation of ferroptosis. Taken together, these findings strongly indicate that both CoA and GSH elevation are essential for the NINJ1 knockdown-mediated ferroptosis protection phenotype.

### NINJ1 physically interacts with xCT

**Fig. 3:**
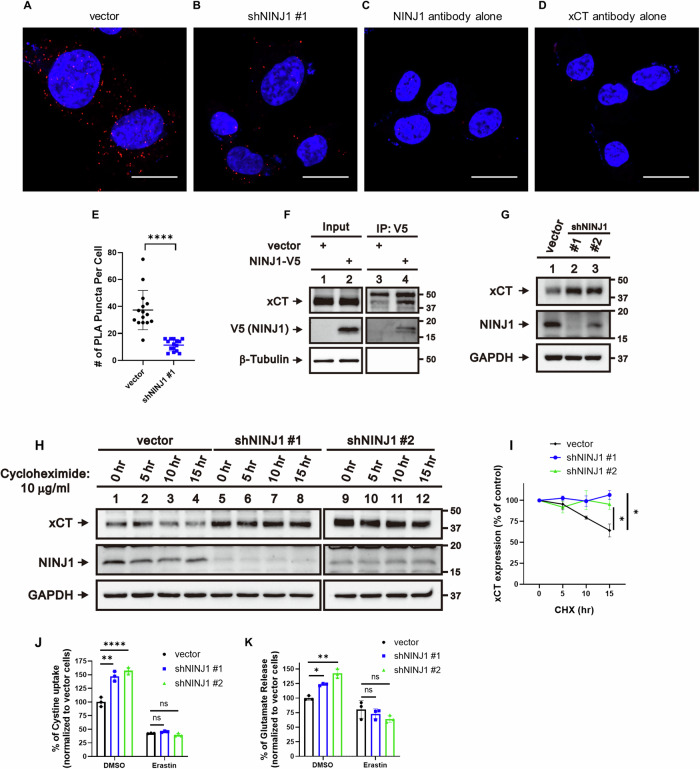
NINJ1 knockdown increased the stability, expression, and activity of xCT. **A**–**E** Control and NINJ1-knockdown HT-1080 cells were fixed in 3.7% paraformaldehyde for 15 min. PLA was subsequently conducted to examine the interaction between xCT and NINJ1. Representative images for the (**A**) vector, (**B**) NINJ1-knockdown, (**C**) NINJ1 antibody alone, and (**D**) xCT antibody alone conditions, along with (**E**) quantification of PLA signals, are presented. Scale bar: 20 μm. **F** Co-IP and Western blot were used to detect the interaction between endogenous xCT and NINJ1-V5. NINJ1 was pulled down using the V5 tag, and the interaction with xCT was detected by Western blot. **G** xCT expression was increased following NINJ1 knockdown in HT-1080 cells, which was verified by Western blot. **H**-**I** Enhanced xCT protein stability post-NINJ1 knockdown in HT-1080 cells was validated by cycloheximide treatment and subsequent Western blot analysis. **J** Cystine uptake levels of control and NINJ1-knockdown HT-1080 cells following erastin treatment (10 μM, 4 h) were determined by Cystine Uptake Assay Kit. **K** Glutamate release levels of control and NINJ1-knockdown HT-1080 cells following erastin treatment (1.25 μM, 20 h) were determined by Amplex® Red Glutamic Acid/Glutamate Oxidase Assay Kit. Error bars in (**E**) and (**I**–**K**) represent SEM (*n* = 15+ for **E**, *n* = 3+ for **I**–**K**).

The CoA synthesis involves the utilization of pantothenate, cysteine, and ATP [50], while GSH is synthesized from cysteine, glutamate, and glycine [57]. Cysteine, a critical component in both CoA and GSH synthesis, is predominantly generated from the cystine imported into cells through the xCT transporter [58]. Since the ferroptosis inhibitory effect of NINJ1 knockdown was specific for the xCT inhibitor, and considering that NINJ1 is also a membrane protein, we investigated its potential relationship with the xCT transporter. First, we determined the potential interaction between endogenous xCT and NINJ1 at the physiological levels using the proximity ligation assay (PLA), which revealed a significant co-localization signal when utilizing two antibodies recognizing endogenous NINJ1 and xCT (Fig. 3A). This interaction was specific, as the NINJ1 knockdown significantly reduced PLA signal and neither NINJ1 nor xCT antibody alone led to significant PLA signals (Fig. 3B–E). The physical association between NINJ1 and xCT was further confirmed using co-immunoprecipitation (Co-IP). In HT-1080 cells expressing NINJ1-V5, NINJ1 was pulled down using the V5 tag, and the co-immunoprecipitated endogenous xCT protein was detected (Fig. 3F). Similarly, using a NINJ1 antibody to pull down endogenous NINJ1, we also detected the associated endogenous xCT (Supplemental Fig. 5A). Next, we employed confocal microscopy to examine their cellular localization. In HT-1080 cells co-expressing EGFP-NINJ1 and mCherry-xCT, we observed colocalization predominantly located on the membrane (Supplemental Fig. 4A–H). In contrast, co-expression of EGFP-NINJ1 with TFRC-mApple showed no similar significant colocalization (Supplemental Fig. 4I–L). Further assessment of EGFP-NINJ1 interactions with endogenous xCT or E-cadherin using specific antibodies revealed a strong merged signal between endogenous xCT and EGFP-NINJ1 (Supplemental Fig. 4M–P), whereas no similar colocalization was observed between E-cadherin and EGFP-NINJ1 (Supplemental Fig. 4Q–T). Together, these findings strongly indicate a potential physical association between NINJ1 and xCT on the plasma membrane.

Since NINJ1 has been reported to mediate PMR in different types of lytic cell death [4, 17, 35], we further assessed the interaction between NINJ1 and xCT under different cell death conditions. Compared to basal conditions, fluorescent confocal imaging revealed no significant changes in the NINJ1-xCT interaction following erastin treatment (Supplemental Fig. 4A–H). Although erastin treatment appeared to increase xCT protein levels, it did not significantly affect the NINJ1-xCT interaction based on the Co-IP result (Supplemental Fig. 5A). Similarly, apoptosis induction in HT-1080 cells using cycloheximide [59] and actinomycin D [60] appeared to reduce xCT protein levels, but neither treatment significantly altered the interaction between NINJ1 and xCT (Supplemental Fig. 5A). These results suggest that NINJ1 interacts with xCT under basal conditions, potentially regulating its activity. Additionally, erastin inhibits xCT activity without disrupting its interaction with NINJ1. During the induction of other forms of cell death, such as apoptosis, NINJ1 may play additional roles, such as mediating PMR [4, 5, 17, 35]. However, its interaction with xCT may not be significantly altered at earlier time points.

### NINJ1 knockdown increased the stability, expression, and activity of xCT

To further determine the functional relevance of NINJ1 regarding xCT activity, we found that NINJ1 knockdown led to a significant upregulation in xCT expression in HT-1080 cells (Fig. 3G). Importantly, the enhanced xCT expression was consistently observed across various cancer cell lines, including MCF7, T47D, CAOV3, and PC3 cell lines (Supplemental Fig. 5B–E), highlighting the broad impact of NINJ1 knockdown. Additionally, NINJ1 knockdown increased xCT stability, potentially contributing to elevated xCT protein levels (Fig. 3H, I). NINJ1 knockdown also increased xCT mRNA levels (Supplemental Fig. 5F), indicating the potential involvement of additional regulatory mechanisms.

Next, we wished to determine whether the increased xCT expression correlates with an enhanced xCT activity. Serving as an antiporter, xCT facilitates intracellular glutamate release and extracellular cystine uptake in a 1:1 ratio [58]. We found that NINJ1 knockdown significantly increased cystine import (Fig. 3J) and glutamate export (Fig. 3K), indicating an enhanced xCT activity. Although such differences were mitigated by erastin treatment, the NINJ1 knockdown group still showed elevated levels of CoA and GSH after erastin treatment (Figs. 2E, F and 3J, K). These results imply that the enhanced xCT function and increased cysteine availability prior to erastin treatment were sufficient to confer protective effects against ferroptosis induced by xCT inhibitors. Together, these data have shown that NINJ1 knockdown increased the levels and function of xCT antiporter.

To further establish the connection between increased xCT expression/activity and the protective effects of NINJ1 knockdown, we overexpressed xCT in HT-1080 cells (Supplemental Fig. 5G). Consistent with the protective phenotype observed in NINJ1 knockdown, xCT overexpression conferred protection against erastin and SAS-induced ferroptosis (Supplemental Fig. 5H, I). Additionally, xCT overexpression increased xCT function (Supplemental Fig. 5J). Even though such differences were also mitigated by erastin treatment, xCT overexpression still exhibited protective effects against erastin-induced ferroptosis (Supplemental Fig. 5H and J). These results further support our hypothesis that the enhanced xCT function and increased cysteine availability before erastin treatment are sufficient to protect cells against ferroptosis induced by xCT inhibitors. Furthermore, xCT overexpression elevated intracellular CoA levels before and after erastin treatment (Supplemental Fig. 5K), further confirming the regulation of CoA levels by xCT activity. Collectively, these results imply that the ferroptotic protective effects of NINJ1 knockdown may be attributed, at least in part, to the enhanced xCT expression and activity, thereby increasing intracellular cystine levels and subsequent elevation of intracellular CoA and GSH.

### NINJ1 overexpression enhanced ferroptosis by downregulating xCT level and activity

**Fig. 4:**
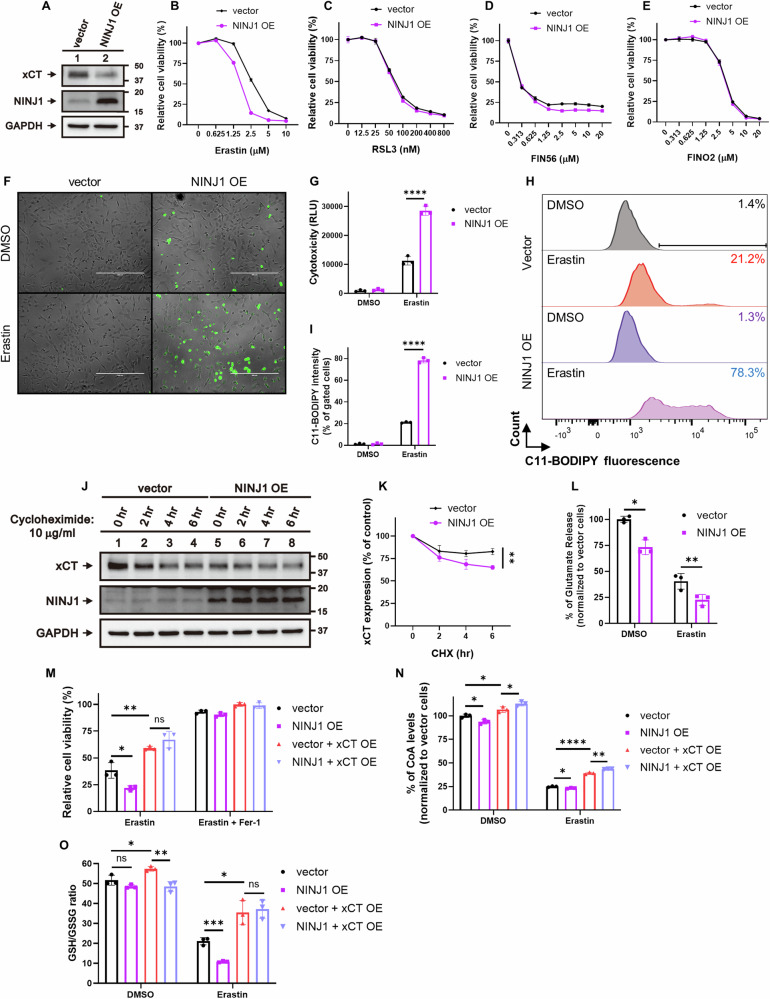
NINJ1 overexpression enhanced ferroptosis by downregulating xCT level and activity. **A** xCT expression was decreased following NINJ1 overexpression in HT-1080 cells, verified by Western blot. **B**–**E** Cell viability of control and NINJ1-overexpressing HT-1080 cells were determined by CellTiter-Glo assay following treatment with indicated concentrations of (**B**) erastin (23 h), (**C**) RSL3 (17 h), (**D**) FIN56 (17 h), and (**E**) FINO2 (23 h). **F**-**G** NINJ1 overexpression enhanced erastin-induced membrane rupture in HT-1080 cells. Membrane rupture of control and NINJ1-overexpressing HT-1080 cells were observed by CellTox Green under fluorescence microscopy (**F**) and quantified (**G**) following erastin treatment (1.25 μM, 21 h). Scale bar: 400 μm. **H**-**I** NINJ1 overexpression enhanced erastin-induced lipid peroxidation in HT-1080 cells. Lipid peroxidation of control and NINJ1-overexpressing HT-1080 cells were determined by C11-BODIPY staining (**H**) and quantified by % of lipid peroxidation positive cells (**I**) following erastin treatment (2.5 μM, 22 h). **J**-**K** Reduced xCT protein stability post-NINJ1 overexpression in HT-1080 cells was validated by cycloheximide treatment and subsequent Western blot analysis. **L** Glutamate release levels of control and NINJ1-overexpressing HT-1080 cells following erastin treatment (1.25 μM, 20 h) were determined by Amplex® Red Glutamic Acid/Glutamate Oxidase Assay Kit. **M** Cell viability in HT-1080 cells with/without NINJ1 or xCT overexpression was determined by CellTiter-Glo assay following treatment with erastin (5 μM) and ferrostatin-1 (Fer-1, 10 μM) for 24 h. **N** Intracellular CoA levels in HT-1080 cells with/without NINJ1 or xCT overexpression were determined by Coenzyme A Assay Kit following erastin treatment (1.25 μM, 24 h). **O** The GSH/GSSG ratios in HT-1080 cells with/without NINJ1 or xCT overexpression were measured by the GSH/GSSG-Glo Assay following erastin treatment (1.25 μM, 24 h). Error bars in (**B**–**E**), (**G**), (**I**), and (**K**–**O**) represent SEM (*n* = 3+).

To further validate our model, we overexpressed NINJ1 in HT-1080 cells to assess its impact on ferroptosis (Fig. 4A). In contrast to the outcomes observed with NINJ1 knockdown, NINJ1 overexpression significantly enhanced ferroptosis induced by erastin (Fig. 4B) while exhibiting no significant impact on other classes of FINs (Fig. 4C–E). NINJ1 overexpression also heightened other erastin-induced molecular features of ferroptosis, including membrane rupture (Fig. 4F–G) and lipid peroxidation (Fig. 4H, I). The NINJ1-mediated ferroptosis-enhancing effect was further confirmed in MDA-MB-231 cells (Supplemental Fig. 6A–D). These results provide additional support for the ability of NINJ1 to regulate ferroptosis by class I FIN but not other classes of FINs. Similarly, NINJ1 overexpression reduced xCT protein expression (Fig. 4A), stability (Fig. 4J–K), and activity (Fig. 4L). NINJ1 overexpression also further reduced CoA levels (Fig. 4N) and the GSH/GSSG ratio (Fig. 4O) following erastin treatment. Interestingly, NINJ1 overexpression increased xCT mRNA levels (Supplemental Fig. 6E), which might indicate a compensatory mechanism that may increase the xCT levels besides affecting its stability.

To further test our hypothesis that the enhanced cell death induced by NINJ1 overexpression is mediated through xCT downregulation, we restored xCT expression by cDNA transduction in HT-1080 cells with NINJ1 overexpression (Supplemental Fig. 6F). As expected, restored xCT expression mitigated the enhanced cell death caused by NINJ1 overexpression (Fig. 4M). Additionally, the reduction in CoA levels and the GSH/GSSG ratio following erastin treatment in NINJ1-overexpressing cells were reversed by xCT restoration (Fig. 4N–O). These results collectively suggest that the enhanced ferroptosis observed with NINJ1 overexpression is mediated by the downregulation of xCT protein level and activity, resulting in a reduction of intracellular CoA and GSH.

We then wish to determine the potential in vivo relevance between NINJ1 and ferroptosis in human cancer. Since NINJ1 was proposed to regulate multiple cell death, we focused on the ferroptosis-biomarkers, such as the induction of CHAC1 and PTGS2 in the TCGA dataset. Our analysis revealed a significant positive correlation between NINJ1 expression and these two ferroptosis markers in the prostate (Supplemental Fig. 6G, H) and rectum adenocarcinoma (Supplemental Fig. 6I, J). This suggests a potential role for NINJ1 in enhancing ferroptosis in these cancer types, consistent with our observation that NINJ1 knockdown protects PC3 cells against erastin-induced ferroptosis (Supplemental Fig. 1G, H). These findings imply a potential therapeutic avenue wherein upregulating NINJ1 could enhance cancer cell response to ferroptosis in these specific cancer types.

## Discussion

**Fig. 5:**
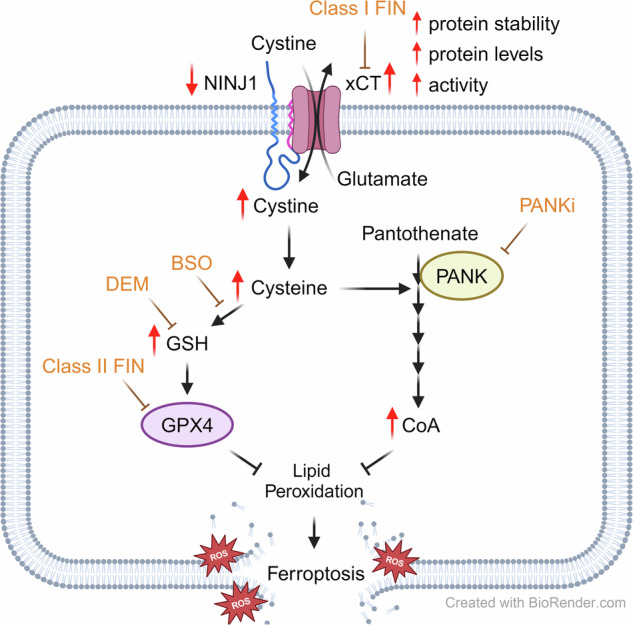
Proposed mechanism of the non-canonical role of NINJ1 in ferroptosis.

Based on these results, we proposed a model illustrating how NINJ1 regulates ferroptosis through a non-canonical mechanism (Fig. 5). Unlike conventional membrane pore formation by aggregated NINJ1 protein that disrupts the membrane, NINJ1 associates with xCT and regulates its level and function. Notably, NINJ1 knockdown enhances xCT level and activity by increasing xCT stability, resulting in elevated CoA and GSH levels, both of which were required to protect ferroptosis. Disruption of either CoA or GSH synthesis abolishes the observed phenotypes of ferroptosis protection. Conversely, NINJ1 overexpression specifically enhanced ferroptosis induced by xCT inhibitors and reduced both xCT levels and activity, resulting in decreased CoA and GSH levels. These findings suggest a potential therapeutic role for NINJ1 in enhancing the sensitivity of cancer cells to ferroptosis.

In our study, we identified a physical association between NINJ1 and xCT, and NINJ1 overexpression was found to decrease the stability, expression, and functionality of xCT. These results suggest the potential role of NINJ1 in regulating xCT degradation and restraining its function. While NINJ1 has been reported to mediate PMR in several types of lytic cell death [4, 17, 35], our findings indicate that neither ferroptosis induction by erastin nor apoptosis induction by cycloheximide or actinomycin D significantly affected the interaction between NINJ1 and xCT. This suggests that NINJ1 has diverse functions: under basal conditions, NINJ1 interacts with xCT to regulate its expression and activity. During different forms of cell death, NINJ1 may play additional roles, such as mediating PMR, although its interaction with xCT may not be significantly altered at earlier time points. Previous studies have shown that the protein stability of the xCT is regulated by various proteins, including solute carrier family 3 member 2 (SLC3A2), CD44 variant (CD44v), MUC1-C, EGFR, and OTUB1. Notably, SLC3A2 is linked with xCT through a disulfide bond, serving as a chaperone to maintain its stability and facilitate proper membrane localization [61]. CD44v, EGFP, and OTUB1 have been reported to interact with and stabilize xCT [62–65], while MUC1-C directly binds to CD44v, promoting xCT stability in the cell membrane [66]. Although we have demonstrated the interaction between NINJ1 and xCT, along with the role of NINJ1 in regulating xCT stability, the specific mechanisms by which this interaction influences xCT stability and activity, as well as the potential involvement of additional proteins in this regulatory process, remain to be determined. Additionally, further investigation is required to pinpoint the exact interaction site between NINJ1 and xCT. It is also crucial to determine whether their binding leads to the dissociation of xCT from other stabilized proteins, thereby promoting xCT instability and triggering endocytosis for degradation, or if it serves as a structural disruptor of xCT.

CoA and its thioester derivatives have been found to participate in various functions, such as the Krebs cycle, amino acid metabolism, fatty acid synthesis, and the regulation of gene expression [67, 68]. In our study, we observed that NINJ1 knockdown led to increased intracellular CoA levels, and the reversal of ferroptosis protection by PANKi validated the role of CoA in the ferroptosis protective effects initiated by NINJ1 knockdown. Previously reported data, along with our recent study in another manuscript, also suggest that CoA acts as a ferroptosis protective agent specifically against class I FINs in various cancer cell lines but does not exhibit the same protective effect against other classes of FINs [24, 36, 37]. This pattern of protection is consistent with the ferroptosis protection associated with NINJ1 knockdown. The increased CoA levels observed in NINJ1 knockdown may result from increased xCT functionality and cystine import, as well as increased PANK1 expression that may increase the de novo synthesis of CoA. Therefore, our results add to the increasing evidence that CoA is an important anti-ferroptosis metabolite subject to various regulators of ferroptosis.

Three recent papers have mentioned the potential role of NINJ1 in ferroptosis across different cell types, including mouse BMDMs, MEFs, and RAW 264.7 cells (a macrophage cell line derived from an Abelson murine leukemia virus-induced tumor) [33–35]. Notably, NINJ1 deficiency did not protect against cell death (as measured by ATP) induced by RSL3 and ML162 [33–35], consistent with our observation that NINJ1 knockdown had no significant impact on RSL3-induced cell death. Additionally, although treatment with erastin and FINO2 induced NINJ1 oligomerization, NINJ1 knockout did not affect the PMR induced by these agents, suggesting that NINJ1 oligomerization may not necessarily trigger NINJ1-dependent PMR [33]. In our study, glycine, known to inhibit NINJ1-mediated PMR in other cell deaths [18], did not influence ferroptosis, indicating that NINJ1 aggregation might not be implicated in the ferroptosis protective effects induced by NINJ1 knockdown. Furthermore, NINJ1 appears to play a role in the later stages of ferroptotic cell death processes (cell lysis/LDH release) [33] and is dispensable for initial steps such as lipid peroxidation, channel-mediated calcium influx, and cell swelling [35]. Although NINJ1 knockout resulted in prolonged protection against cell lysis, it did not affect cell death induced by GPX4 inhibitors. Notably, the reduction in cell lysis due to NINJ1 deficiency was significant only in BMDMs and MEFs, with the minimal effect observed in RAW 264.7 cells [35], indicating a cell type-dependent role of NINJ1 in ferroptosis that may vary between normal and cancer cells. The onset of cancer often triggers a multitude of cellular alterations, including altered metabolism and aberrant cell signaling [69]. Therefore, it is conceivable that NINJ1 may assume a distinct or additional role in ferroptosis within the context of cancer. Our study predominantly focuses on unraveling the involvement of NINJ1 in ferroptosis in cancer, presenting a model that deviates from the traditional role of NINJ1 in mediating PMR during lytic cell death. Furthermore, the association between NINJ1 and the xCT transporter, along with the availability of GSH and CoA, could also imply a potential mechanism underlying the tumor suppression function of NINJ1 in certain cancer types.

While we analyzed ferroptosis in various cancer cell lines commonly used in ferroptosis research treated with different classes of FINs, it is possible that NINJ1 may play different roles in ferroptosis of other cell types and under other ferroptosis-triggering stimuli, such as nutrient deprivation, oxidative stresses, and ischemia-reperfusion [70, 71]. Although our current research primarily focuses on the involvement of CoA and GSH, it is also plausible that additional mechanisms and metabolites may also contribute to ferroptosis protection. Additionally, while our research centers on cancer cells, since previous studies on NINJ1 were mainly performed in macrophages, especially in BMDMs [4, 5, 17, 35], it will be valuable to further validate our results using BMDMs with NINJ1 knockout. Consequently, there is still much to be explored regarding the role of NINJ1 in the ferroptosis of different cell types and under various ferroptosis-inducing conditions and disease processes.

Ferroptosis has been appreciated to have significant implications for human diseases [21, 22]. For example, ferroptosis mediates cell death during acute kidney injury, stroke, cardiomyopathy [72], and various neurodegenerative diseases. Therefore, our study on the role of NINJ1 in ferroptosis suggests that modulating NINJ1, a membrane-accessible protein, holds a significant therapeutic potential. For example, various NINJ1 neutralization antibodies have been reported [17, 73, 74], and their application in these ferroptosis-associated diseases could potentially mitigate ferroptosis by inhibiting NINJ1. In human cancer, numerous studies highlight the crucial role of ferroptosis in tumor suppression and suggest its therapeutic potential in triggering tumor elimination and restraining tumor growth. For example, rapidly proliferating cancer cells may be more sensitive to ferroptosis given their upregulated intracellular iron levels [31], abnormal lipid metabolism, and increased ROS production [75, 76]. Moreover, several processes associated with treatment resistance and metastasis, including epithelial-mesenchymal transition (EMT) [31], ECM signaling [30], and activation of YAP/TAZ [77], can promote ferroptosis [23]. Therefore, targeting ferroptosis may provide important therapeutic options for tumors resistant to current treatments. In this context, various approaches for activating NINJ1 may have the potential to enhance ferroptosis and sensitize tumor cells to ferroptosis-inducing therapies.

## Methods

### Cell Culture

The HT-1080, MDA-MB-231, PC3, MCF7, T47D, and CAOV3 cells employed in this study were cultured following standard conditions in a 37 °C humidified incubator with 5% CO_2_. The cell lines were authenticated through STR DNA profiling and confirmed to be mycoplasma-free before being cryopreserved by the Cell Culture Facility. The culture media used were Dulbecco’s Modified Eagle Medium (DMEM) (GIBCO-11995) for HT-1080, MDA-MB-231, MCF7, T47D, and CAOV3, and Ham’s F-12K (Kaighn’s) Medium (GIBCO-21127022) for PC3. Additionally, all media were supplemented with 10% heat-inactivated FBS (Sigma, # F0926) and 10,000 UI/ml streptomycin and penicillin (ThermoFisher, #15140122).

### Constructs and lentivirus viral infections

To induce NINJ1 knockdown, we purchased small interfering RNAs (siRNAs) targeting human NINJ1 RNA from Dharmacon (D-017671-01-04), and two distinct shRNAs targeting human NINJ1 RNA were acquired from Sigma (TRCN0000063769, TRCN0000289088). For overexpression studies, pLX304-NINJ1 cDNA with a V5 tag was sourced from DNASU (HsCD00436123 and HsCD00438752), pWPI-SLC7A11 cDNA with an HA tag from Addgene (#201643), and pCDH-TFRC-mApple cDNA from Addgene (#179384). By using Gateway cloning, NINJ1 cDNA from pENTR223-NINJ1 (DNASU, HsCD00505254) and SLC7A11 cDNA from pENTR223-SLC7A11 (DNASU, HsCD00512940) were individually subcloned into pLVpuro-CMV-N-EGFP (Addgene, #122848) and pLVpuro-CMV-N-mCherry (Addgene, #123221) for lentiviral expression. Lentiviruses carrying specific constructs were generated by transfecting HEK-293T cells with a mixture of the lentiviral vector, pMD2.G, and psPAX2 at a ratio of 1:1:0.1 using TransIT-LT1 transfection reagent (Mirus). The resulting blend was then filtered through a 0.45 µm cellulose acetate membrane (VWR, #28145-481). Subsequently, target cells were infected with 250 µl of lentivirus-containing media and polybrene (8 µg/ml), and subjected to puromycin, blasticidin, or G418 selection.

### Chemicals and focused compound screen

Erastin (Bio-techne, 5449); SAS (MedchemExpress, HY-14655); RSL3 (Cayman, 19288); FIN56 (Cayman, 25180); FINO2 (MedchemExpress, HY-129457); PANKi (Cayman, 31002); BSO (Sigma, B2515); DEM (Sigma, D97703); Fer-1 (Selleckchem, S7243); etomoxir sodium salt (Selleckchem, S8244); lovastatin (Selleckchem, S2061); TOFA (Selleckchem, S6690); dorsomorphin (Selleckchem, S7306); alisertib (Selleckchem, S1133); verdinexor (Cayman, 26171); leptomycin B (Cayman, 10004976); tipifarnib (MedchemExpress, HY-10502); methotrexate (Selleckchem, S1210); pitstop2 (Sigma, SML1169); EML425 (Selleckchem, S2977); C646 (Selleckchem, S7152); brequinar (Selleckchem, S3565); elamipretide (Selleckchem, S9803), cycloheximide (Sigma, C7698), actinomycin D (Sigma, A9415).

The focused compound screen is presented in Fig. 2A–D and Supplemental Fig. 2. Each cell viability graph was generated individually using the CellTiter-Glo assay following treatment with the indicated concentrations of erastin, with or without the specified compounds. Detailed treatment concentrations and durations are provided in Fig. 2B–D and Supplemental Fig. 2. The relative viability percentages for cells treated with 5 μM erastin, with or without the indicated compounds, were compiled from each graph and summarized in the heatmap shown in Fig. 2A.

### Cell viability and cytotoxicity

The CellTiter-Glo luminescent cell viability assay (Promega) was used to measure cell viability according to the manufacturer’s instructions. After the treatment, 10 μl of CellTiter-Glo reagent was applied to each well (containing 100 µl of media), followed by shaking and a 10 min incubation. Luminescence was then quantified using a chemiluminescence plate reader, and relative cell viability was calculated as a percentage of control (DMSO)-treated cells. Cytotoxicity quantification was performed using the CellTox Green assay (Promega) and a fluorescence plate reader following the manufacturer’s protocol, with the dye added to the media at a 1:1000 dilution.

### Lipid peroxidation assay

Lipid peroxidation was measured using C11-BODIPY (ThermoFisher Scientific, D3861) staining based on the manufacturer’s protocols. In brief, at the end of treatments, cells were incubated with 10 µM C11-BODIPY dye for 1 h. Following harvest, washing, and resuspension in PBS with 1% BSA, lipid peroxidation levels were evaluated and quantified through flow cytometry (FACSCanto TM II, BD Biosciences).

### Western blots and co-immunoprecipitation

Cells were lysed in RIPA buffer with protease inhibitor (Roche, 04693159001), vortexed continuously at 4 °C for 30 min, and centrifuged at 16,000 rpm for 10 min. The supernatants were collected, and protein concentrations were determined using the Pierce BCA protein assay kit (ThermoFisher, #23225). For western blotting, proteins were loaded onto 12–15% SDS-PAGE gels, transferred to a PVDF membrane, and blocked for 1 h in 5% non-fat milk in 1x TBST. Membranes were then incubated overnight at 4°C with the following primary antibodies: NINJ1 (1:1000, Invitrogen, PA5-95755), GAPDH (1:1000, Cell Signaling, #97166), PANK1 (1:1000, Cell Signaling, #23887), xCT (1:1000, Cell Signaling, #12691), V5 tag (1:1000, Cell Signaling, #13202), and β-tubulin (1:1000, Cell Signaling, #2128).

For the Co-IP experiment, NINJ1 was isolated from HT-1080 cells, with or without NINJ1-V5 overexpression, using either a NINJ1 antibody (Invitrogen, PA5-95755) or the V5-tagged Protein Purification Kit Ver.2 (MBL, 3317). For purification of endogenous NINJ1 or NINJ1-V5, HT-1080 cells were lysed in NP-40 buffer with protease inhibitors, followed by constant vortexing at 4 °C and centrifugation. The supernatant was then purified using Dynabeads™ Protein G (Invitrogen, 10004D) or the V5-tagged Protein Purification Kit (MBL, 3317), according to the manufacturer’s instructions. Western blot analysis was performed to detect the associated proteins.

### Quantitative real-time PCR

By utilizing the RNeasy Mini Kit (Qiagen), RNA extraction was based on the manufacturer’s protocol. cDNA reverse transcription was conducted utilizing SuperScript IV reverse transcriptase (Invitrogen) and random hexamers. The synthesized cDNA was mixed with Power SYBR Green PCR Mix (Applied Biosystems) and specific primers, following the manufacturer’s protocols. Quantitative real-time PCR reactions were performed on a StepOnePlus Real-time PCR system (Applied Biosystems). The provided data represents three independent biological repetitions, with gene expression normalized to GAPDH levels. The primer sequences used were as follows: Human PANK1 primers - sense, 5’- TGG AAC GCT GGT TAA ATT GGT -3’, antisense, 5’- CCC AGT TTT CCC ATA AGC AGT AT -3’; Human SLC7A11 primers - sense, 5’- TCT CCA AAG GAG GTT ACC TGC -3’, antisense, 5’- AGA CTC CCC TCA GTA AAG TGA C -3’; Human GAPDH primers - sense, 5’- GGA GCG AGA TCC CTC CAA AAT -3’, antisense, 5’- GGC TGT TGT CAT ACT TCT CAT GG -3’.

### Intracellular CoA levels

Intracellular CoA levels were quantified using the Coenzyme A Assay Kit (Abcam, ab138889) and a fluorescent plate reader, following the manufacturer’s instructions. The results are presented as relative CoA levels, calculated as a percentage relative to control (DMSO)-treated vector cells.

### GSH/GSSG ratio

To assess the GSH/GSSG ratio, the GSH/GSSG-Glo Assay (Promega) was performed following the manufacturer’s protocols. In brief, after the designated treatment period, cells in the 96-well plate were washed with PBS and treated with 50 μl of either total GSH lysis reagent (for total GSH measurement) or oxidized GSH lysis reagent (for GSSG measurement). Following this, 50 µl of Luciferin Generation Reagent was added to each sample and incubated for 30 min. Subsequently, 100 μl of Luciferin Detection Reagent was applied, and samples were incubated for an additional 15 min before luminescence was measured. The GSH/GSSG ratios were calculated from luminescence measurements, expressed in relative light units (RLU), using the formula (total GSH RLU-GSSG RLU)/(GSSG RLU/2).

### Proximity Ligation Assay

The interaction between NINJ1 and xCT proteins was assessed using the Duolink Proximity Ligation Assay (Sigma) following the manufacturer’s protocol. Initially, cells were fixed with 3.7% formaldehyde, permeabilized using 0.3% Triton X-100, and blocked with Duolink Blocking Solution for 1 h. Primary antibodies targeting NINJ1 (1:200, Invitrogen, PA5-95755) and xCT (1:200, Invitrogen, MA5-44922) were applied to each sample and incubated overnight at 4 °C. Subsequent to washing with 1× Wash Buffer A, PLUS and MINUS PLA probes were introduced to each sample and incubated at 37 °C for 1 h. Following additional washes, ligation solution was administered and allowed to incubate at 37 °C for 30 min. After three additional washes, amplification solution was added and incubated at 37 °C for 100 min. Following two washes with 1× Wash Buffer B and one wash with 0.1x Wash Buffer B, the slides were mounted using Duolink PLA Mounting Medium with DAPI for nuclear staining and then covered with a coverslip. Immunofluorescence microscopy was performed with a confocal microscope equipped with Airyscan for super-resolution quality (880, Zeiss).

### Immunofluorescence confocal microscopy

After the indicated treatment, EGFP-NINJ1-overexpressing HT-1080 cells, with or without mCherry-xCT or TFRC-mApple overexpression, were fixed with 3.7% formaldehyde, permeabilized using 0.3% Triton X-100, and blocked with 5% BSA for 1 h. The samples were then incubated with primary antibodies for 1 h, followed by secondary antibody incubation for an additional hour. The slides were mounted using DAPI Fluoromount-G (SouthernBiotech) with DAPI for nuclear staining and then covered with a coverslip. Immunofluorescence microscopy was performed using a confocal microscope equipped with Airyscan for super-resolution imaging (Zeiss 880). The antibodies used were xCT (1:100, Invitrogen, MA5-44922), E-cadherin (1:100, BD, 610404), and Goat anti-mouse IgG Alexa Fluor 594 (1:500, Invitrogen, A-1100).

### Cystine uptake assay

xCT activity was evaluated using the Cystine Uptake Assay Kit (DOJINDO, UP05) following the manufacturer’s protocol. In summary, after completing the treatment, cells in a 96-well plate underwent three washes with cystine-free and serum-free medium, followed by incubation in cystine-free and serum-free medium at 37 °C for 5 min. Subsequently, cystine uptake solution (with DMSO or erastin) or cystine-free and serum-free medium (blank) was added to each sample and incubated at 37 °C for 30 min. After three washes with PBS, methanol and the working solution were added to each sample, thoroughly mixed, and then incubated for an additional 30 min at 37 °C. The fluorescence intensity of each sample was measured using a fluorescence plate reader. The fluorescent signal was normalized to the total cell number, and cystine uptake levels were expressed as a percentage relative to control (DMSO)-treated vector cells.

### Glutamate release assay

The release of intracellular glutamate into the extracellular medium was quantified using the Amplex Red Glutamic Acid/Glutamate Oxidase Assay Kit (Invitrogen, A12221) following the manufacturer’s protocol. Specifically, upon completing the treatment, cells in 6-well plates were subjected to two washes with PBS and then incubated in Na^+^-containing and glutamine-free media containing either DMSO or erastin for 1 h. Subsequently, 50 μl of the medium from each well was transferred to a 96-well plate and incubated with 50 μl of a reaction mixture for 30 min. The fluorescence intensity of each sample was then measured using a fluorescence plate reader. After normalizing the fluorescent signal to the total cell number at the end of the experiment, glutamate release levels were expressed as a percentage relative to control (DMSO)-treated vector cells.

### Statistics

Bar graphs include individual data points to represent biological replicate numbers, while figure legends for line graphs specify the number of biological replicates. Data are expressed as mean ± standard error of the mean (SEM). Statistical analyses were performed using a two-tailed Student’s *t*-test, one-way ANOVA with Tukey’s multiple comparisons, or two-way ANOVA with Dunnett’s multiple comparisons in IBM SPSS Statistics version 21 or GraphPad Prism. Error bars indicate SEM, and significance between samples is indicated as follows: *p < 0.05, **p < 0.01, ***p < 0.001, and ****p < 0.0001.

## Supplementary information

Supplemental Figures

Full and uncropped western blots

## Data Availability

All data supporting the findings of this study are available from the authors upon reasonable request.

## Materials availability

All reagents supporting the findings of this study are available from the authors upon reasonable request.
