# Supplementary material for: NINJ1 regulates ferroptosis via xCT antiporter interaction and CoA modulation
Source: Cell Death Dis. 2024 Oct 18;15(10):755. doi: 10.1038/s41419-024-07135-1 (PMC11489787; doi:10.1038/s41419-024-07135-1)
Supplement: Supplementary file 1 — Supplemental Figures [file 41419_2024_7135_MOESM1_ESM.pdf]

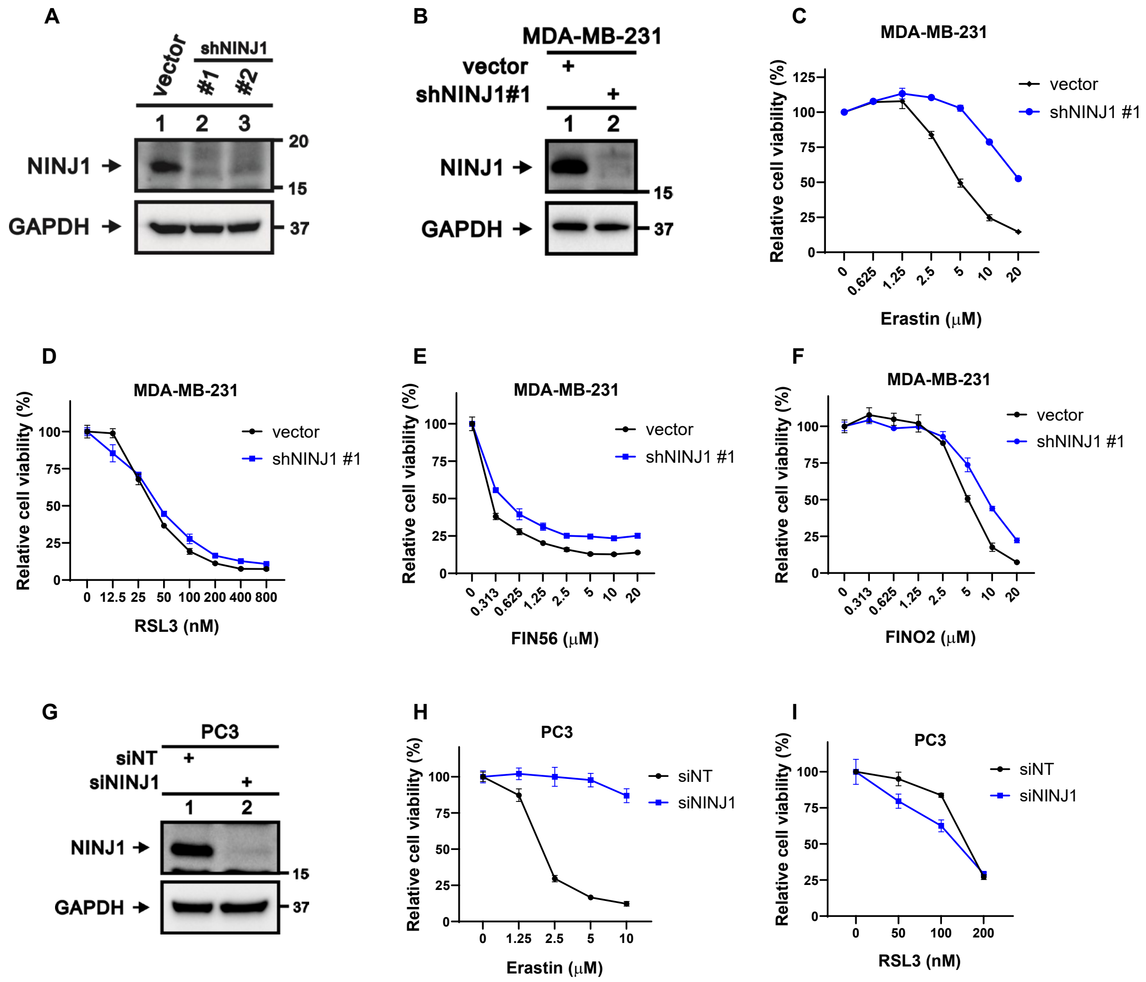

Supplemental Figure 1. (A) Two NINJ1 shRNA significantly reduced the expression of NINJ1 in HT-1080 cells verified by Western blot. (B) NINJ1 shRNA #1 significantly reduced the expression of NINJ1 in MDA-MB-231 cells verified by Western blot. (C-F) Cell viability of control and NINJ1-knockdown MDA-MB-231 cells were determined by CellTiter-Glo assay following treatment with indicated concentrations of (C) Erastin (26 h), (D) RSL3 (21 h), (E) FIN56 (21 h), and (F) FINO2 (26 h). (G) NINJ1 siRNA significantly reduced the expression of NINJ1 in PC3 cells verified by Western blot. (H-I) Cell viability of control and NINJ1-knockdown PC3 cells were determined by CellTiter-Glo assay following treatment with indicated concentrations of (H) Erastin (28 h) and (I) RSL3 (28 h). Error bars in (C-F) and (H-I) represent SEM (n = 3+).

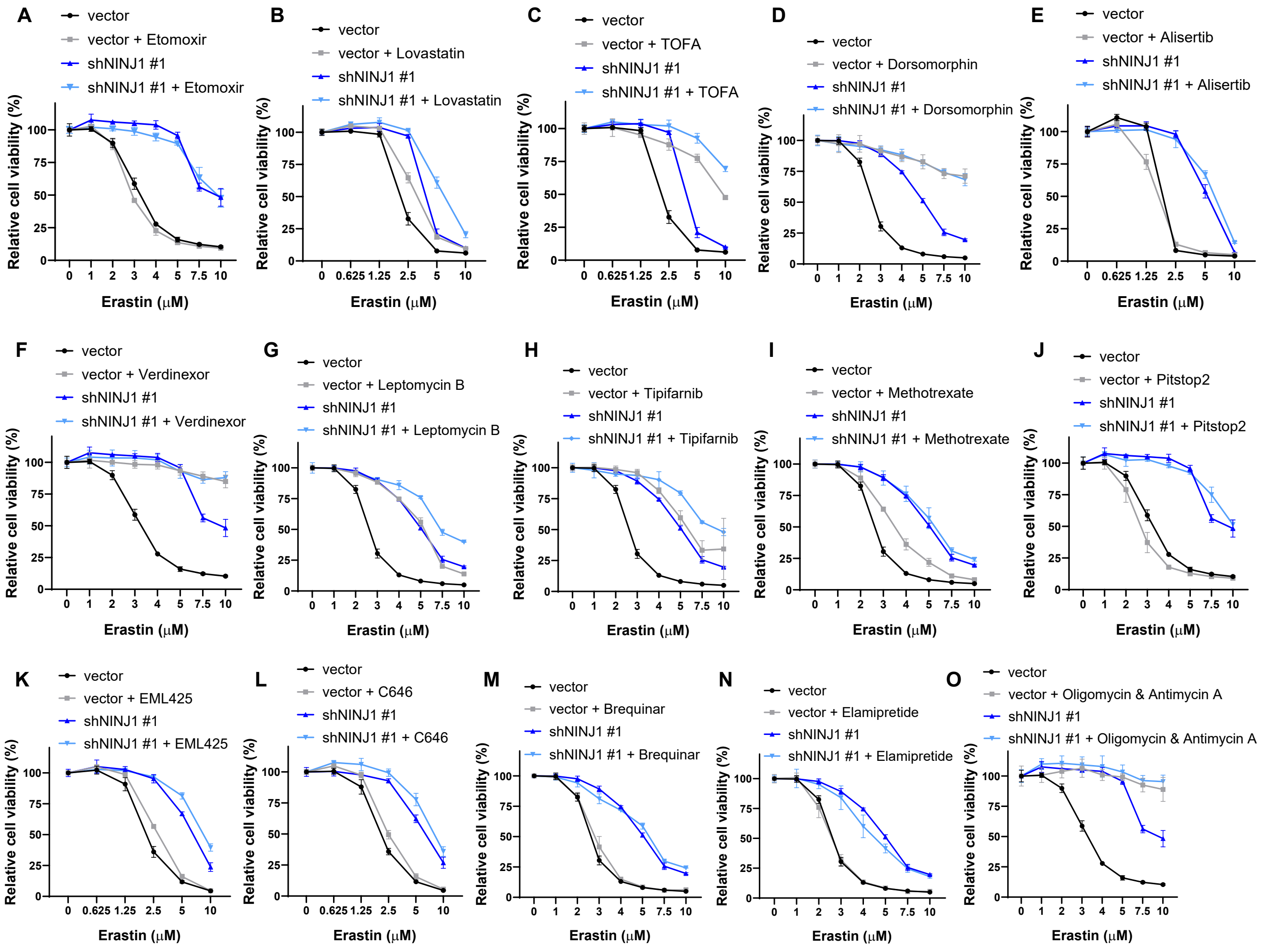

Supplemental Figure 2. (A-O) Cell viability of control and NINJ1-knockdown HT-1080 cells were determined by CellTiter-Glo assay following treatment with indicated concentrations of Erastin combined with/without (A) β-oxidation inhibitor (Etomoxir 5 μM, 18 h), (B) HMGCR inhibitor (Lovastatin 10 μM, 22 h), (C) ACC inhibitor (TOFA 25 μM, 22 h), (D) AMPK inhibitor (Dorsomorphin 10 μM, 18 h), (E) Aurora kinase A inhibitor (Alisertib 10 μM, 21 h), (F-G) XPO1/CRM1 inhibitors (F Verdinexor, 18 h & G Leptomycin B 25 μg/ml, 18 h), (H) farnesyltransferase inhibitor (Tipifarnib 10 μM, 18 h), (I) dihydrofolate reductase inhibitor (Methotrexate 2 μM, 18 h), (J) clathrin-mediated endocytosis inhibitor (Pitstop2 25 μM, 18 h), (K-L) p300/CBP Inhibitor (K EML425 5 μM, 23 h & L C646 5 μM, 23 h), (M) DHODH inhibitors (Brequinar 500 μM, 18 h), (N) mitochondrial-targeted peptide (Elamipretide 20 μM, 18 h), and (O) mitophagy induction (the combination treatment of Oligomycin 10 μM and Antimycin A 10 μM, 18 h). (A, F, J, O) were performed at the same time. (B, C) were performed at the same time. (D, G-I, M, N) were performed at the same time. Each group mentioned above has the same control and NINJ1-knockdown HT-1080 cells following treatment with indicated concentrations of Erastin alone. Error bars in (A-O) represent SEM (n = 3+).

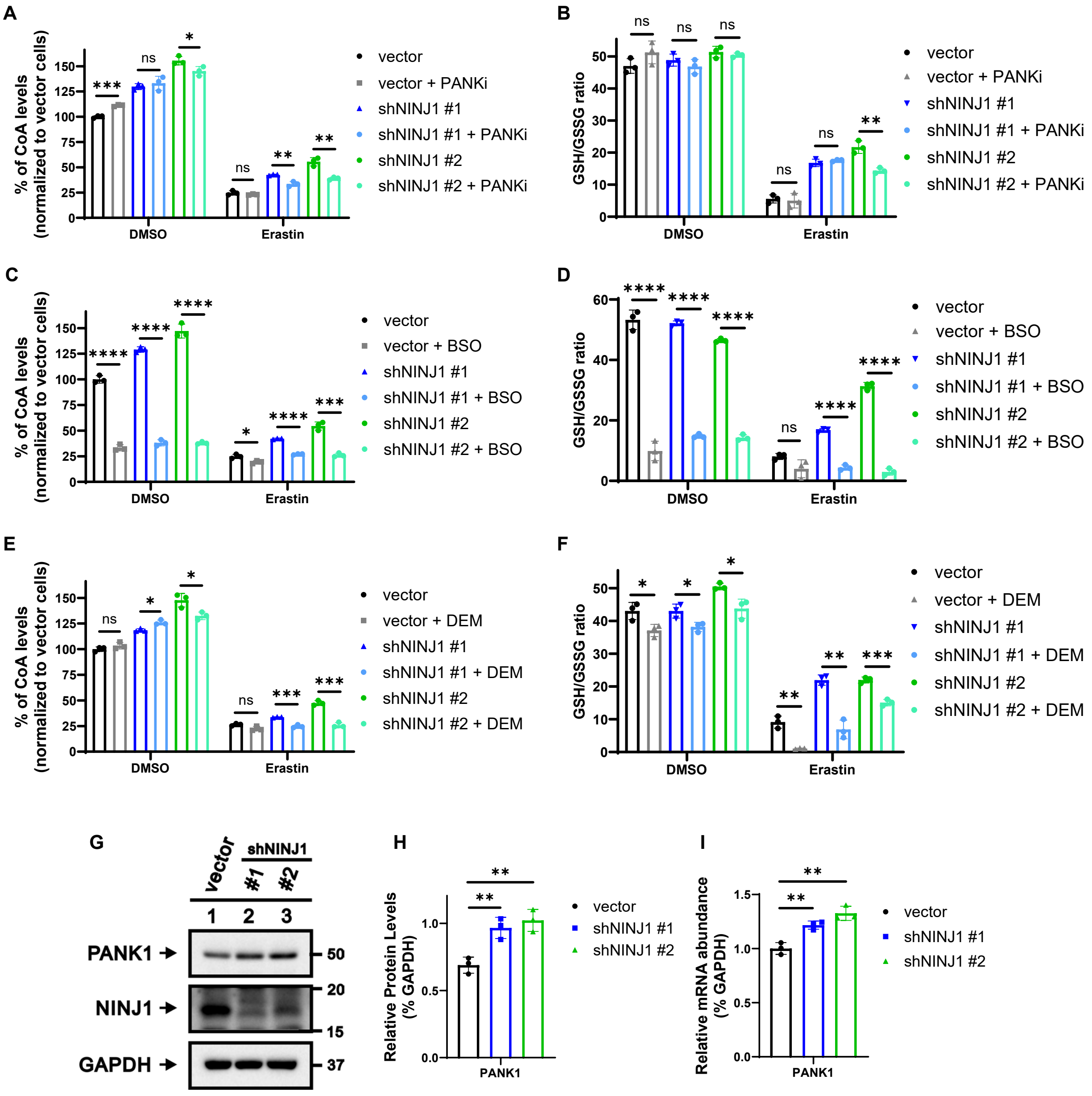

Supplemental Figure 3. (A, C, E) Intracellular CoA levels in control and NINJ1-knockdown HT-1080 cells were determined by Coenzyme A Assay Kit following treatment with Erastin (1.25 µM) in combination with either (A) pantothenate kinase inhibitor (PANKi, 5 µM), (C) buthionine sulfoximine (BSO, 500 µM), or (E) diethylmaleate (DEM, 200 µM) for 24 h. (B, D, F) The GSH/GSSG ratios in control and NINJ1-knockdown HT-1080 cells were measured by the GSH/GSSG-Glo Assay following treatment with Erastin (1.25 µM) in combination with either (B) pantothenate kinase inhibitor (PANKi, 5 µM), (D) buthionine sulfoximine (BSO, 500 µM), or (F) diethylmaleate (DEM, 200 µM) for 24 h. (G-I) PANK1 expressions were increased following NINJ1 knockdown in HT-1080 cells verified by (G-H) Western blot and (I) Quantitative real-time PCR. Error bars in (A-F) and (H-I) represent SEM (n = 3+).

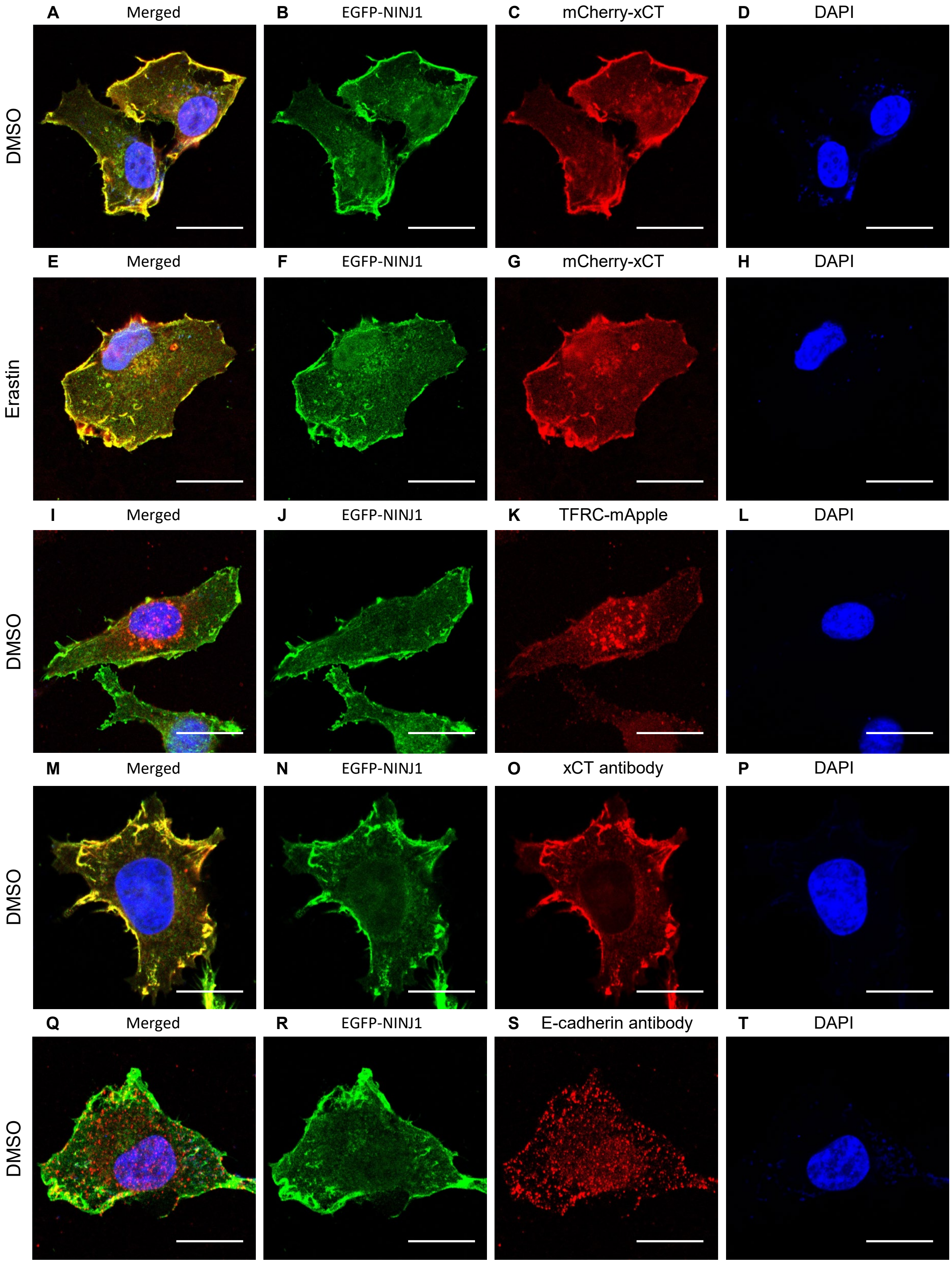

Supplemental Figure 4. (A-H) EGFP-NINJ1 and mCherry-xCT were co-overexpressed in HT-1080 cells following treatment with (A-D) DMSO and (E-H) Erastin (5 µM, 24 h). Fluorescent images of (A and E) merged, (B and F) EGFP-NINJ1 alone, (C and G) mCherry-xCT alone, and (D and H) DAPI are presented. (I-L) EGFP-NINJ1 and TFRC-mApple were co-overexpressed in HT-1080 cells. Fluorescent images of (I) merged, (J) EGFP-NINJ1 alone, (K) TFRC-mApple alone, and (L) DAPI are presented. (M-P) HT-1080 cells with EGFP-NINJ1 overexpression were stained with an xCT antibody. Fluorescent images of (M) merged, (N) EGFP-NINJ1 alone, (O) xCT antibody staining alone, and (P) DAPI are presented. (Q-T) HT-1080 cells with EGFP-NINJ1 overexpression were stained with an E-cadherin antibody. Fluorescent images of (Q) merged, (R) EGFP-NINJ1 alone, (S) E-cadherin antibody staining alone, and (T) DAPI are presented. Scale bar for (A-T): 20 µm.

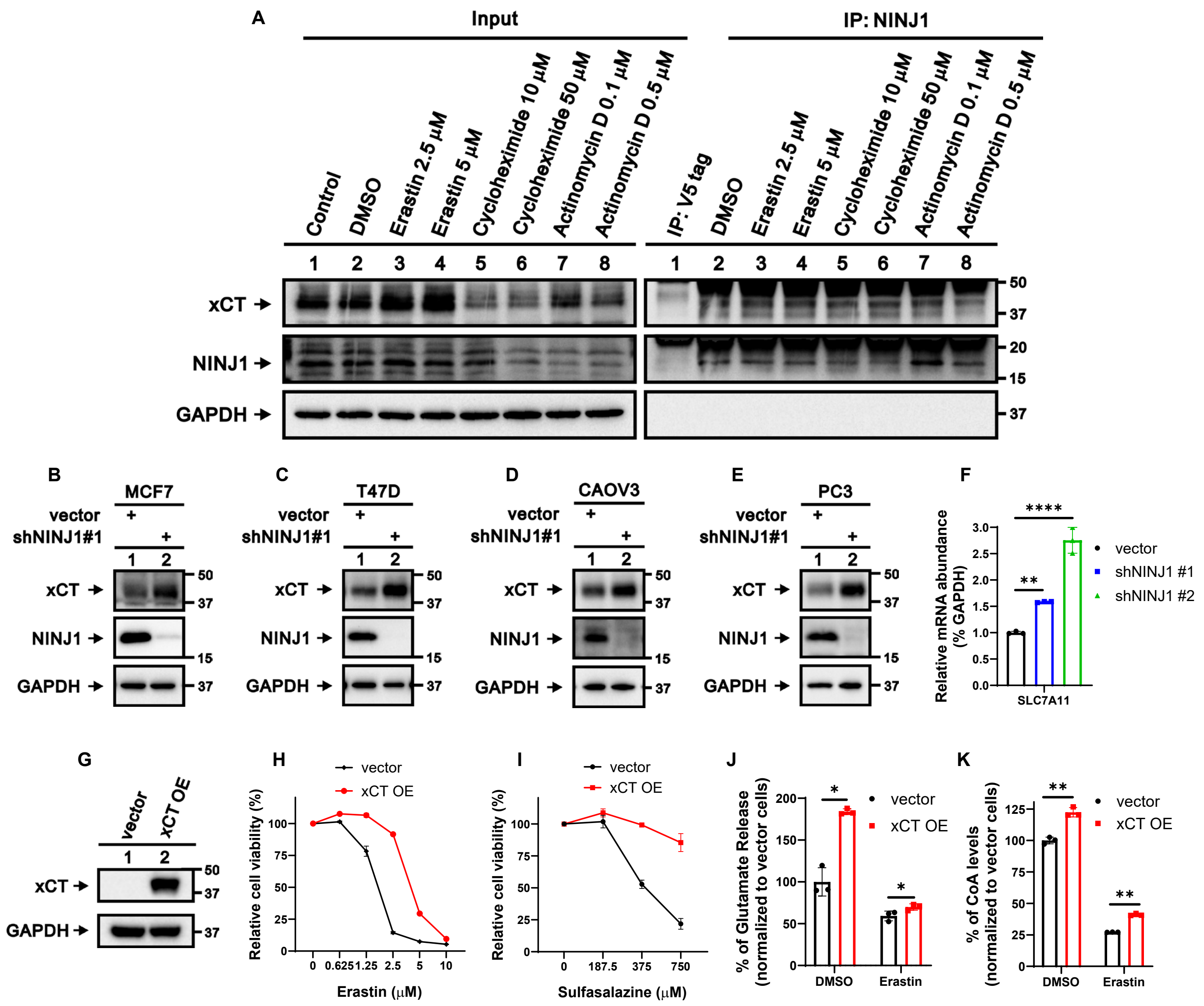

Supplemental Figure 5. (A) CO-IP and Western blot were used to detect the interaction between endogenous xCT and NINJ1 under basal conditions and upon induction of different cell death. Cells were treated with the indicated concentrations of Erastin (ferroptosis induction), cycloheximide (apoptosis induction), or actinomycin D (apoptosis induction) for 24 h. NINJ1 was pulled down using either a V5 tag antibody (negative control) or a NINJ1-specific antibody, and the interaction with xCT was detected by Western blot. (B-E) xCT expressions were increased following NINJ1 knockdown in (B) MCF7, (C) T47D, (D) CAOV3, and (E) PC3 cells verified by Western blot. (F) SLC7A11 mRNA expressions were increased following NINJ1 knockdown in HT-1080 cells verified by Quantitative real-time PCR. (G) xCT overexpression efficacy in HT-1080 cells was verified by Western blot. (H-I) Cell viability of control and xCT-overexpressing HT-1080 cells were determined by CellTiter-Glo assay following treatment with indicated concentrations of (H) Erastin (24 h) and (I) Sulfasalazine (24 h). (J) Glutamate release levels in the control and xCT-overexpressing HT-1080 cells following Erastin treatment (1.25 μM, 20 h) were determined by Amplex® Red Glutamic Acid/Glutamate Oxidase Assay Kit. (K) Intracellular CoA levels in control and xCT-overexpressing HT-1080 cells following Erastin treatment (1.25 μM, 24hr) were determined by Coenzyme A Assay Kit. Error bars in (F) and (H-K) represent SEM (n = 3+).

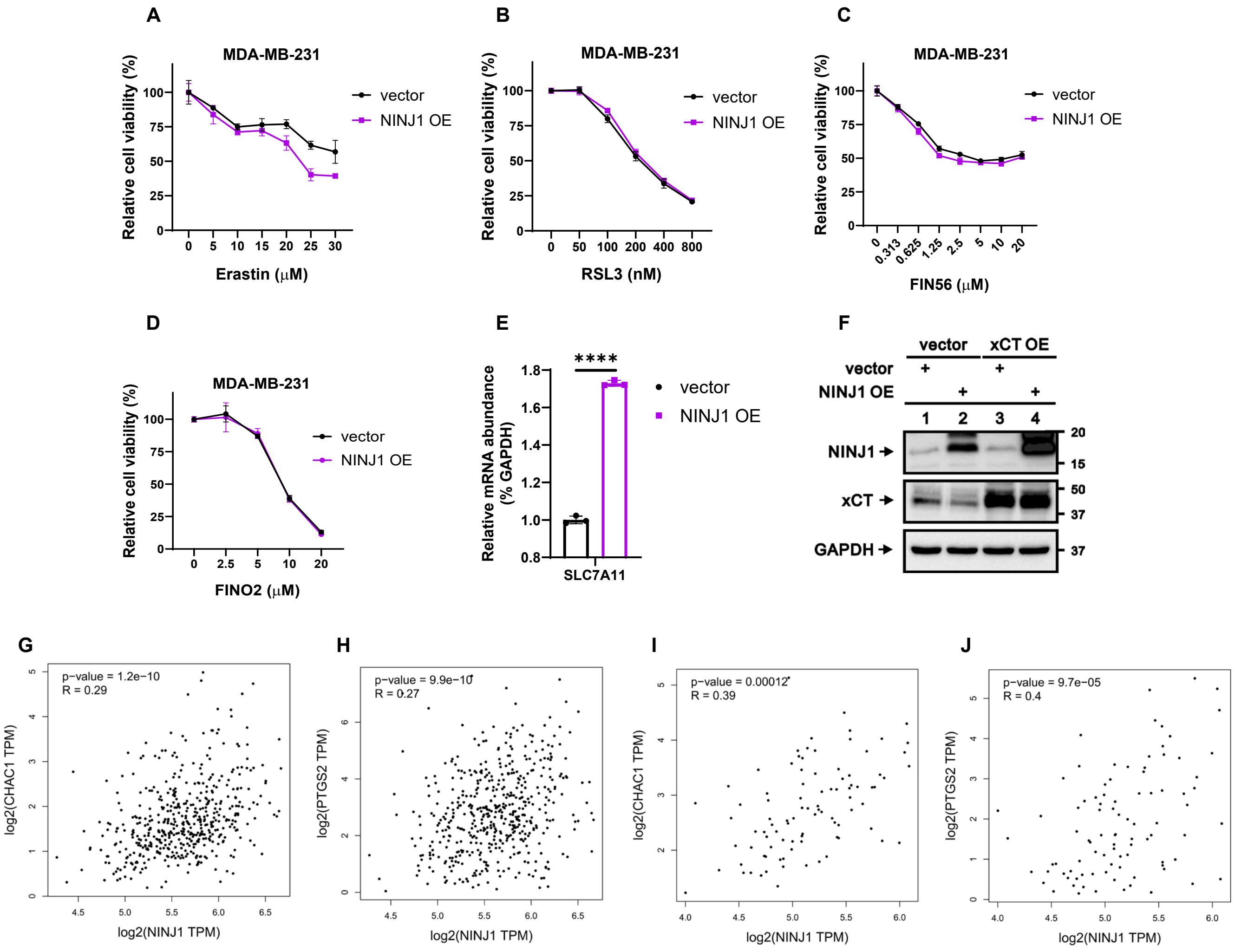

Supplemental Figure 6. (A-D) Cell viability of control and NINJ1-overexpressing MDA-MB-231 cells were determined by CellTiter-Glo assay following treatment with indicated concentrations of (A) Erastin (57 h), (B) RSL3 (26 h), (C) FIN56 (26 h), and (D) FINO2 (32 h). (E) SLC7A11 mRNA expressions were increased following NINJ1 overexpression in HT-1080 cells verified by Quantitative real-time PCR. (F) NINJ1 and xCT overexpression efficacy in HT-1080 cells were verified by Western blot. (G-H) The correlation of NINJ1 with ferroptosis markers (G) CHAC1 and (H) PTGS2 in human prostate adenocarcinoma dataset from TCGA. (I-J) The correlation of NINJ1 with ferroptosis markers (I) CHAC1 and (J) PTGS2 in the human rectum adenocarcinoma dataset from TCGA. The results for (G-J) are based upon data generated by the TCGA Research Network: https://www.cancer.gov/tcga. Error bars in (A-E) represent SEM (n = 3+).
