# Supplementary material for: NINJ1 regulates ferroptosis via xCT antiporter interaction and CoA modulation
Source: Cell Death Dis. 2024 Oct 18;15(10):755. doi: 10.1038/s41419-024-07135-1 (PMC11489787; doi:10.1038/s41419-024-07135-1)

Figure 3

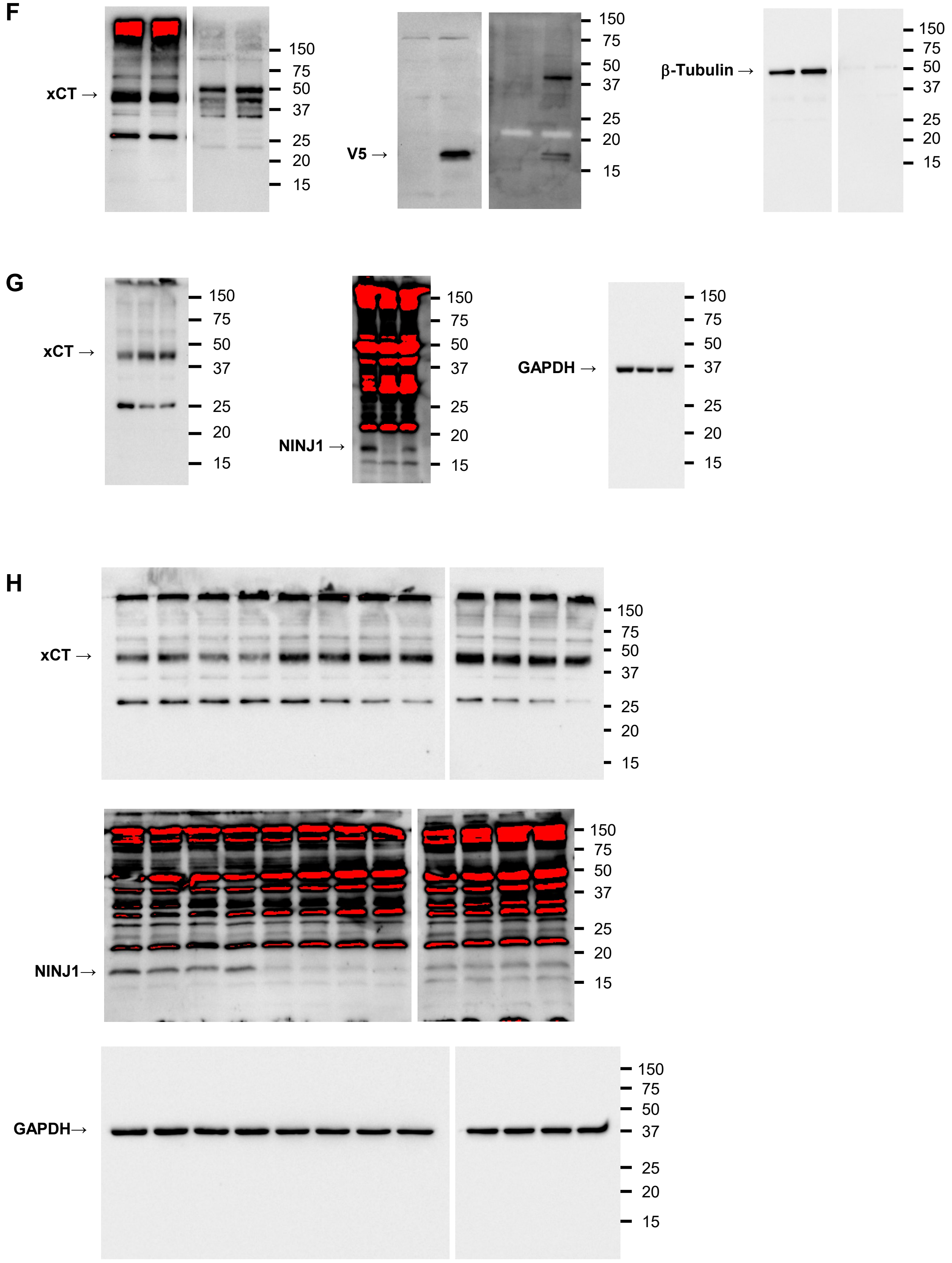
F
xCT →
150
75
50
37
25
20
15
V5 →
150
75
50
37
25
20
15
β-Tubulin →
150
75
50
37
25
20
15
G
xCT →
150
75
50
37
25
20
15
NINJ1 →
150
75
50
37
25
20
15
GAPDH →
150
75
50
37
25
20
15
H
xCT →
150
75
50
37
25
20
15
NINJ1→
150
75
50
37
25
20
15
GAPDH→
150
75
50
37
25
20
15

Figure 4

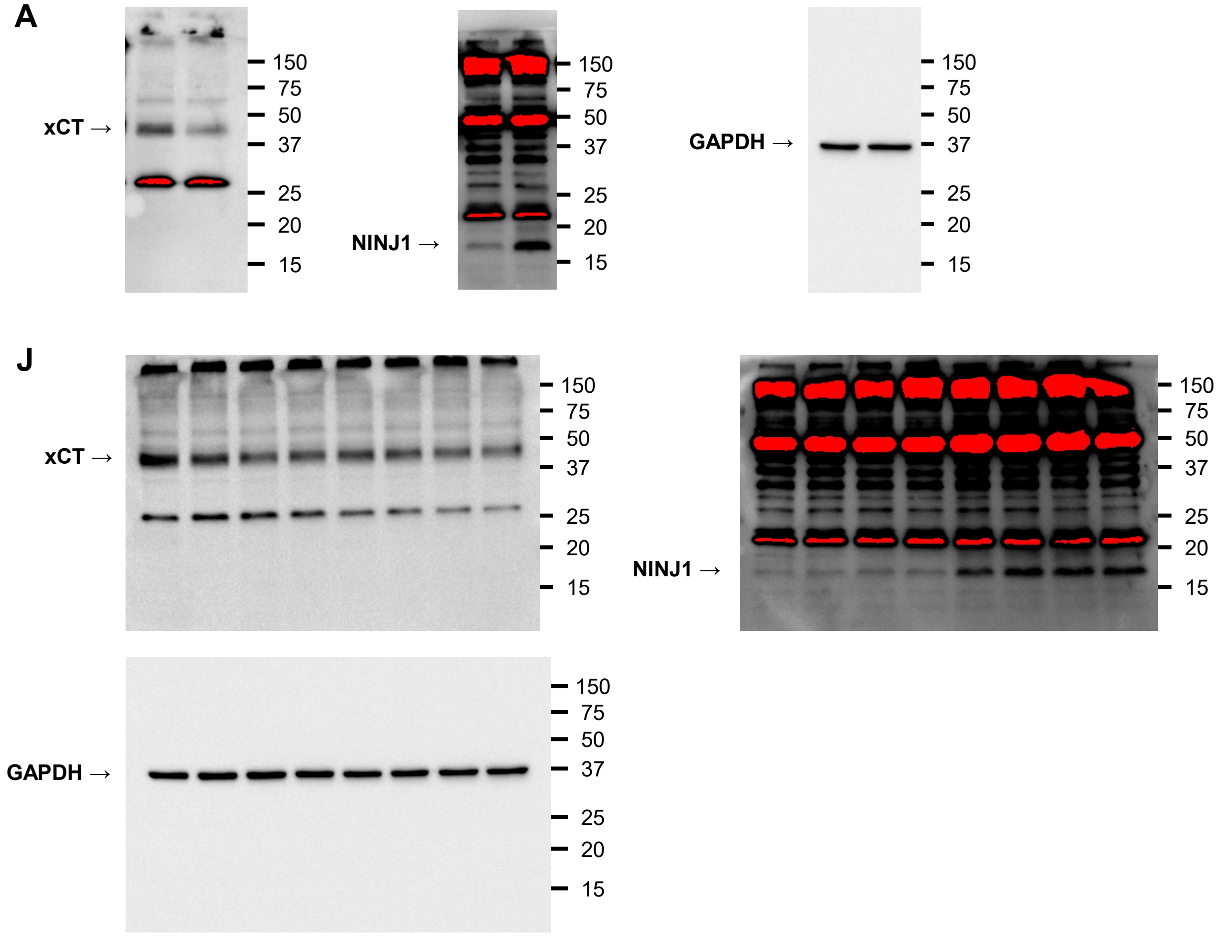

Supplemental Figure 1

A

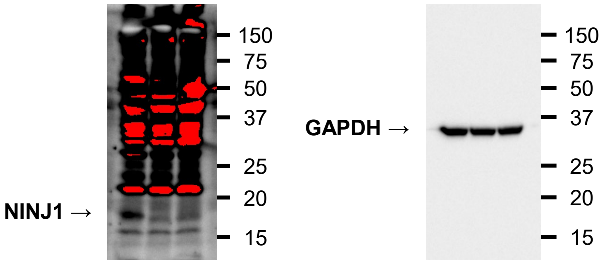

B

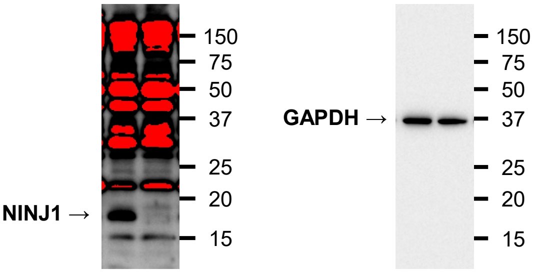

G

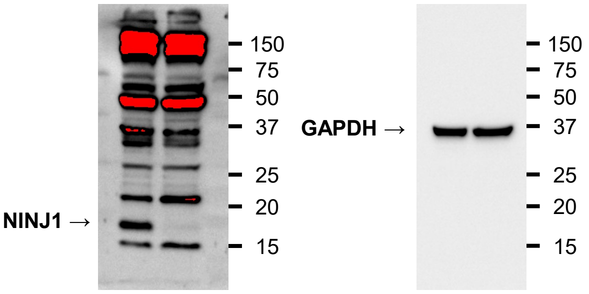

Supplemental Figure 3

G

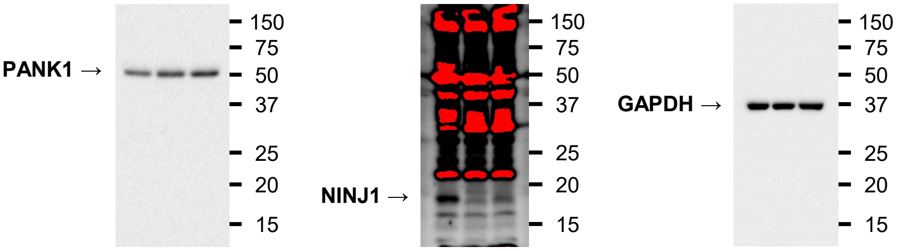

Supplemental Figure 5

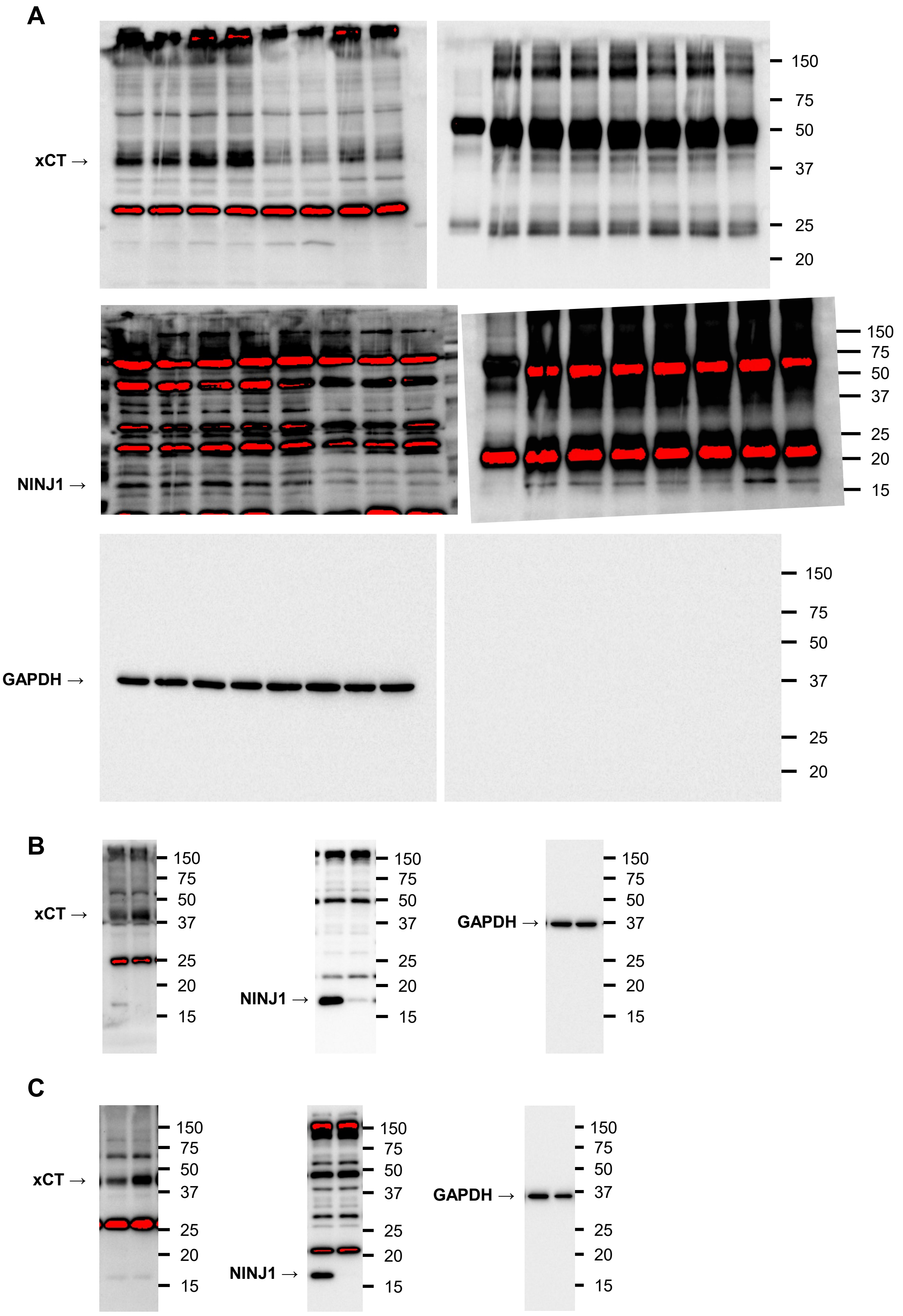

Supplemental Figure 5

D

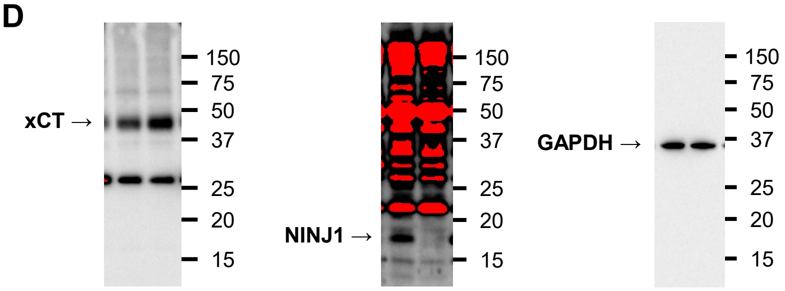

E

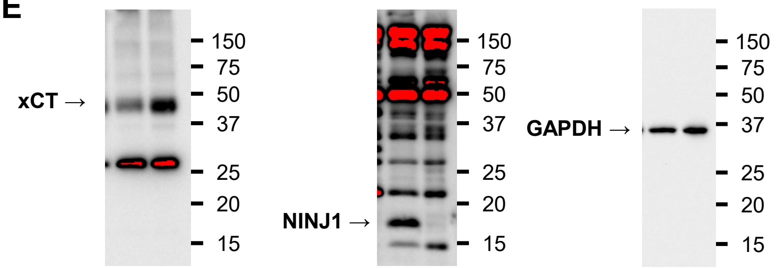

G

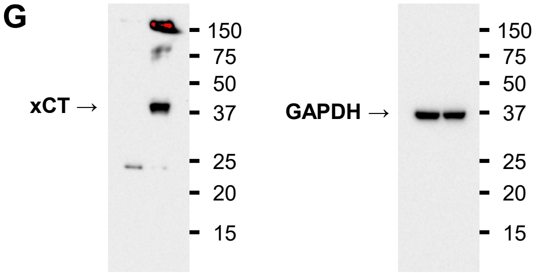

Supplemental Figure 6

F

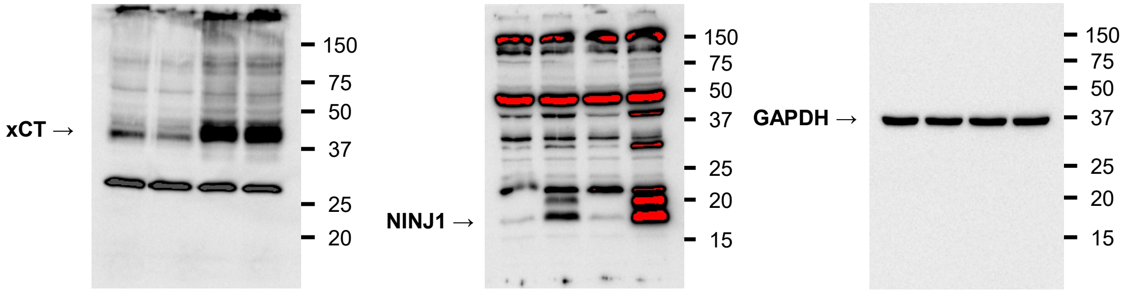

Supplement: Supplementary file 2 — Full and uncropped western blots [file 41419_2024_7135_MOESM2_ESM.pdf]
